# Corticosteroid-refractory checkpoint inhibitor pneumonitis in lung cancer: biological heterogeneity and mechanism-informed management

**DOI:** 10.3389/fimmu.2026.1983756

**Published:** 2026-10-28

**Authors:** Rong Zhang, Xiaoxue Zhang, Xiulan He, Min Xiang

**Affiliations:** 1 Department of Respiratory and Critical Care Medicine, West China Tianfu Hospital, Sichuan University, Chengdu, China; 2 Department of Respiratory and Critical Care Medicine, West China Hospital, Sichuan University, Chengdu, Sichuan, China

**Keywords:** checkpoint inhibitor pneumonitis, corticosteroid-refractory pneumonitis, immune checkpoint inhibitors, immune-related adverse events, immunosuppressive therapy, lung cancer, precision medicine

## Abstract

Immune checkpoint inhibitors (ICIs) have transformed the treatment of lung cancer, but checkpoint inhibitor pneumonitis (CIP) remains a potentially life-threatening immune-related adverse event. A subset of patients fails to improve despite adequate glucocorticoid therapy and develops corticosteroid-refractory CIP, which is associated with respiratory failure, substantial mortality, and considerable therapeutic uncertainty. This review critically examines the clinical definition, phenotypic characteristics, immunological mechanisms, and therapeutic evidence for corticosteroid-refractory CIP. Emerging data suggest that refractoriness should not be regarded as a uniform failure of glucocorticoid treatment, but rather as a biologically heterogeneous and dynamically evolving syndrome involving persistent tissue-resident T-cell activation, inflammatory T-cell–myeloid crosstalk, cytokine-network redundancy, innate immune dysregulation, and, in some patients, transition toward maladaptive tissue repair and structural lung remodeling. Current second-line treatments, including mycophenolate mofetil, infliximab, intravenous immunoglobulin, tocilizumab, cyclophosphamide, and calcineurin inhibitors, are supported predominantly by retrospective studies and case-report and case-series evidence, with no established optimal treatment sequence. We therefore propose a hypothesis-generating phenotype-to-therapy framework that distinguishes lymphocyte-dominant, cytokine-dominant, fulminant acute lung injury/diffuse alveolar damage, innate/myeloid-dominant, and organizing/fibrotic-remodeling phenotypes while recognizing their potential overlap and temporal evolution. Translating this framework into clinical practice will require harmonized definitions, longitudinal multimodal phenotyping, treatment-predictive biomarkers, and prospective comparative studies. Ultimately, management should move beyond empirical escalation of immunosuppression toward identifying the dominant biological process at a given disease stage and matching treatment intensity and mechanism accordingly.

## 1 Introduction

Immune checkpoint inhibitors (ICIs) have transformed the treatment of multiple malignancies, particularly advanced lung cancer. By targeting the programmed cell death protein 1/programmed death-ligand 1 (PD-1/PD-L1) axis or cytotoxic T-lymphocyte-associated antigen 4 (CTLA-4), they relieve immunosuppression within the tumor microenvironment and reinvigorate antitumor T-cell responses (1–3). In non-small cell lung cancer (NSCLC), pembrolizumab, nivolumab, atezolizumab, and durvalumab are used in first-line, consolidation, and perioperative settings with significant survival gains (1, 2), and pivotal trials including KEYNOTE-024, CheckMate-017/057, and IMpower133 confirmed prolonged survival in NSCLC and extensive-stage small cell lung cancer (3–6). With widespread adoption, however, immune-related adverse events (irAEs) have become a major factor limiting ICI use and may affect virtually every organ system (7); among these, checkpoint inhibitor pneumonitis (CIP) is one of the most severe, owing to its insidious onset, rapid progression, and potentially fatal course (8, 9).

Guidelines from the American Society of Clinical Oncology (ASCO), the European Society for Medical Oncology (ESMO), and the National Comprehensive Cancer Network (NCCN) provide a severity-graded framework for the management of CIP (10–12). Glucocorticoids are the first-line treatment for CIP (10–12), and most patients respond to adequate corticosteroid therapy, with reported response rates of approximately 70%-80% (13). A proportion respond poorly or relapse during tapering, manifesting as a corticosteroid-refractory or corticosteroid-dependent course (14, 15); these patients typically have more severe disease, often requiring intensive care, and their mortality risk is markedly increased, making early recognition and effective management a pressing clinical problem (16).

Corticosteroid-refractory CIP presents several challenges: no internationally standardized definition exists, and differing criteria across guidelines and studies affect trial eligibility and cross-study comparability; prognosis is poor, with a high risk of respiratory failure and death; evidence for second-line and salvage therapy derives largely from case reports and small retrospective series, with no strategy supported by randomized controlled trials (RCTs); and the mechanisms of disease onset and glucocorticoid resistance remain incompletely defined, involving persistent T-cell activation, cytokine imbalance, and innate immune dysregulation (17, 18).

This review integrates current clinical and translational evidence to characterize corticosteroid-refractory CIP, critically examines candidate mechanisms of glucocorticoid refractoriness and available rescue therapies, and proposes a phenotype-to-therapy framework for transitioning from empirical immunosuppressive escalation toward mechanism-informed precision management.

## 2 Definition and clinical features of corticosteroid-refractory CIP

### 2.1 Current definitions

The ASCO, ESMO, and NCCN guidelines use broadly similar response-based approaches to identify corticosteroid-refractory CIP, although no universally standardized definition has been established. ASCO recommends treatment escalation when no clinical improvement is observed after approximately 48 hours of appropriate glucocorticoid therapy (11), whereas ESMO recommends escalation when no improvement is observed after 72 hours (10). NCCN similarly recommends reassessment within 48 hours for grade 3–4 CIP and consideration of additional immunosuppression if no improvement occurs (19). Adequate glucocorticoid therapy is defined as guideline-concordant systemic treatment matched to disease severity, generally prednisone or an equivalent at 1–2 mg/kg/day for grade 2 CIP and intravenous methylprednisolone at 1–2 mg/kg/day for grade 3–4 CIP, with response assessed over approximately 48–72 hours. Accordingly, we define corticosteroid-refractory CIP as persistent or worsening clinical or respiratory manifestations despite approximately 48–72 hours of guideline-concordant systemic glucocorticoid therapy, after appropriate evaluation for infection, tumor progression, and other alternative causes of deterioration.

Corticosteroid-refractory CIP should be distinguished from corticosteroid-dependent, recurrent, and chronic CIP. In this review it refers to persistent deterioration or lack of meaningful improvement despite adequate glucocorticoid therapy within the guideline response-assessment window, typically 48-72h (10–12, 15). Corticosteroid-dependent CIP initially improves but relapses during tapering or cannot be weaned, which may overlap therapeutically with refractory disease without indicating primary glucocorticoid failure (14, 15, 20). Recurrent CIP denotes a new episode after previous improvement or resolution, including recurrence after ICI re-exposure (21, 22), whereas chronic CIP has been defined as pneumonitis persisting or worsening during tapering after ICI discontinuation and requiring immunosuppression for at least 12 weeks (23). Notably, a multicenter longitudinal analysis by Zhou et al. independently identified a chronic phase beginning at approximately 13 weeks after CIP onset, broadly consistent with this temporal threshold, although their cohort was not restricted to corticosteroid-refractory disease (24). Pooling refractory, dependent, recurrent, and chronic phenotypes can therefore obscure differences in disease biology, treatment timing, and outcomes.

### 2.2 Incidence and mortality

The reported incidence of CIP varies with treatment regimen, tumor type, and study population: among patients with NSCLC receiving ICIs, clinical trial data indicate approximately 3.0%-6.3% and real-world estimates approach 13.0%-19.0% (25, 26), and dual checkpoint blockade may increase risk relative to monotherapy (8, 27). Approximately 10% or more of patients respond poorly to glucocorticoids and some progress to refractory disease requiring additional immunosuppression (25, 28). Prognosis is poor, with a high risk of respiratory failure and death. Reported mortality rates are heterogeneous, but outcomes are consistently worse than in corticosteroid-sensitive disease, and available evidence derives primarily from single-center retrospective studies and case series; Beattie et al. reported a 90-day all-cause mortality/hospice referral of 50% among patients treated with second-line immunosuppression (29).

Beyond short-term mortality, available longitudinal evidence suggests that CIP may follow a temporally evolving clinical course. In a multicenter retrospective study of 56 patients with CIP, Zhou et al. proposed three phases according to time from CIP onset: an acute phase (≤4.9 weeks), a subacute phase (4.9-13.1 weeks), and a chronic phase (≥13.1 weeks) (24). Across these phases, symptoms, performance status, and CIP grade generally decreased, while radiological inflammatory lesions progressively resolved. However, structural abnormalities persisted in a subset of patients, including traction bronchiectasis in 3 of 11 patients reaching the chronic phase. Histopathological findings also suggested evolution from predominantly acute fibrinous pneumonitis in the acute phase toward organizing pneumonia in the subacute phase and fibrotic remodeling at later stages. Circulating IL-6, IL-10, and hsCRP likewise decreased over time. Importantly, this cohort included patients with CIP irrespective of glucocorticoid responsiveness and therefore does not establish the natural history or long-term prognosis of corticosteroid-refractory CIP specifically. Nevertheless, these findings provide a useful temporal framework indicating that persistent disease may evolve from an inflammation-dominant process toward organizing or fibrotic remodeling, underscoring the need for longitudinal reassessment in patients with an apparently refractory course.

### 2.3 Clinical presentation

#### 2.3.1 Symptoms

Patients with corticosteroid-refractory CIP typically present with dyspnea, cough, fever, and hypoxemia, and some deteriorate rapidly (8, 9). Compared with patients who remit after glucocorticoid therapy, they more often develop persistent hypoxemia and acute respiratory failure and more frequently require intensive care and mechanical ventilation (28, 29). Continued deterioration despite adequate therapy may indicate ongoing immune-mediated inflammatory activation and should prompt escalated immunosuppression (11).

#### 2.3.2 Computed tomography features

High-resolution computed tomography (HRCT) is essential for the diagnosis, radiological classification, and longitudinal assessment of CIP (30, 31). CIP is radiologically heterogeneous; common patterns include organizing pneumonia (OP), non-specific interstitial pneumonia (NSIP), hypersensitivity pneumonitis (HP), and diffuse alveolar damage (DAD), with OP generally most frequent (18, 30, 31), and in a recent cohort of 102 patients an OP pattern accounted for approximately 50% of cases (32). CT patterns may correlate with severity: the DAD or acute interstitial pneumonia pattern typically shows extensive bilateral ground-glass opacities and consolidation with septal thickening and traction bronchiectasis, indicating widespread alveolar–capillary barrier disruption, and is more frequent in high-grade CIP and associated with rapidly progressive respiratory failure and poorer outcomes (18, 31).

#### 2.3.3 Laboratory findings

No laboratory parameter specifically predicts corticosteroid-refractory CIP. Some patients show elevated C-reactive protein (CRP), lactate dehydrogenase, and neutrophil counts with lymphopenia. Lung injury-associated markers such as Krebs von den Lungen-6 (KL-6) and surfactant protein D may reflect parenchymal damage and have ancillary diagnostic value in ICI-related pneumonitis, although their capacity to predict glucocorticoid responsiveness requires validation (33), and serial KL-6 monitoring awaits prospective confirmation (34). Pro-inflammatory cytokines such as interleukin-6 (IL-6) are elevated in some patients, yet no single mediator predicts treatment failure, and although tocilizumab has shown potential value in selected irAEs and in a small number of corticosteroid-refractory CIP cases (35), treatment selection and response prediction remain highly uncertain (16, 29).

#### 2.3.4 Bronchoalveolar lavage fluid features

Bronchoalveolar lavage (BAL) is an important ancillary investigation, principally to obtain lower respiratory tract specimens and exclude pulmonary infection, alveolar hemorrhage, and tumor progression; it may be considered in grade 2 or higher disease, particularly when infection cannot be excluded or imaging is atypical (10, 11). No single bronchoalveolar lavage fluid (BALF) parameter can confirm or exclude CIP. BALF frequently demonstrates lymphocytosis, although findings are heterogeneous: Chen et al. reported a mean lymphocyte proportion of 36.7% ± 22.5% in 42 patients, with 81.0% meeting a lymphocytic pattern (>15%) and no difference by disease grade (36), whereas Guo et al. found higher proportions associated with high-grade disease and relapse during tapering (21). Because these studies are single-center, small, and retrospective, BALF lymphocyte proportions cannot reliably assess severity or prognosis.

Lymphocyte subsets are similarly heterogeneous: Chen et al. found relative CD8^+^ predominance, with 73.8% of patients having a CD4^+^/CD8^+^ ratio below 1 (36), whereas Guo et al. found a ratio above 1 associated with recurrence during corticosteroid tapering (21). Comparable changes occur in drug-induced lung injury, hypersensitivity pneumonitis, and other interstitial lung diseases and therefore lack specificity (37, 38), and BALF neutrophilia cannot distinguish infection from immune-mediated injury (10, 11).

BALF cytokines have been explored as candidate biomarkers with inconsistent results: Kowalski et al. found significantly elevated BALF IL-6 and group-dependent elevations in BALF IL-17A, with BALF IL-6 showing potential discriminatory value for CIP (39); Si et al. reported increased IP-10/CXCL10 and CD3^+^CD8^+^ T-cell proportions without differences in IL-6 (40); and Ploch et al. observed elevated BALF IL-6 in CIP and other immune-mediated conditions, although only three patients with CIP were included (41). These inconsistencies may reflect differences in case definitions, disease stage, sampling, and assay platforms. Single-cell sequencing has further shown BALF T-cell enrichment, cytotoxic T-cell activation, and expansion of pro-inflammatory monocytes/macrophages in CIP (42).

Overall, BALF data support the concept that corticosteroid-refractory CIP is unlikely to represent a single inflammatory phenotype. Available cohorts suggest potentially distinct lymphocyte-dominant, T-cell subset-skewed, and myeloid/neutrophil-enriched patterns, accompanied by variable cytokine signatures involving IL-6, IL-17A, and CXCL10/IP-10. However, these putative phenotypes remain poorly defined because most published BALF studies enrolled heterogeneous CIP populations and were not designed to distinguish corticosteroid-responsive from corticosteroid-refractory disease. Small sample sizes, retrospective designs, variable timing of bronchoscopy relative to corticosteroid exposure, inconsistent exclusion of infection, and differences in cytokine assays further limit comparability. Thus, BALF cellular and cytokine profiling is currently best viewed as a research tool for mechanistic stratification rather than a validated method for defining corticosteroid-refractory CIP subgroups or guiding rescue therapy.

### 2.4 Risk factors

Identifying patients at risk of progressing to corticosteroid-refractory CIP is important for early risk stratification, but existing evidence largely concerns the risk of CIP or of severe CIP rather than predictors of glucocorticoid treatment failure (10, 11). Pre-existing interstitial lung disease or interstitial lung abnormalities, pulmonary fibrosis, and combination immunotherapy may increase risk (43), as may previous thoracic radiotherapy (44, 45), whereas advanced age, smoking history, and chronic obstructive pulmonary disease have been implicated inconsistently (43, 46). Although the reported incidence of CIP is relatively high in NSCLC (47), coexisting smoking-related lung injury, interstitial abnormalities, and prior radiotherapy make it uncertain whether NSCLC histology is an independent risk factor. These factors principally reflect susceptibility to CIP rather than validated predictors of glucocorticoid failure.

The single-center retrospective study by Li et al. is among the few to address corticosteroid-refractory CIP directly, comparing 28 refractory with 38 corticosteroid-sensitive patients (16). Refractory patients more frequently had pre-existing interstitial lung disease, higher severity grades, higher neutrophil counts and procalcitonin, and lower albumin; on multivariate analysis, grade 3–4 disease at diagnosis and a higher absolute neutrophil count were independently associated with refractoriness (16). These factors nevertheless require prospective multicenter validation.

## 3 Mechanisms of corticosteroid-refractory checkpoint inhibitor pneumonitis

Corticosteroid-refractory CIP is likely a biologically heterogeneous syndrome rather than a single mechanistic entity, involving persistent tissue-resident T-cell activation, inflammatory T-cell–myeloid crosstalk, cytokine and chemokine amplification (48–50). In some patients, failure of epithelial repair and structural remodeling may also contribute to persistent lung injury (51, 52). Because most mechanistic studies have compared patients with and without CIP rather than steroid-sensitive and steroid-refractory disease, these pathways should be regarded as candidate mechanisms of refractoriness rather than established determinants of glucocorticoid resistance (16, 53). Retrospective cohorts indicate that refractory disease is associated with greater initial severity, elevated peripheral neutrophil counts, and poor long-term outcomes, and pre-existing pulmonary abnormalities have been associated with severe CIP (16, 17, 53).

### 3.1 Sustained adaptive immune activation

#### 3.1.1 Persistent CD8^+^ T-cell activation, tissue residency, and dysfunctional differentiation

Sustained T-cell activation may contribute to corticosteroid-refractory CIP by maintaining localized inflammatory circuits that are incompletely suppressed by glucocorticoids: by blocking PD-1/PD-L1 or CTLA-4 signaling, ICIs promote effector T-cell proliferation, cytotoxicity, and clonal expansion, and once this activation becomes locally entrenched within the lung parenchyma, inflammation may become relatively refractory to conventional immunosuppression (25).

Single-cell RNA and TCR sequencing have provided critical insights into the antigen-associated immunopathology of CIP. Zhang et al. identified in bronchoalveolar lavage fluid (BALF) a predominance of CD8^+^ T cells bearing effector memory and tissue-resident memory (Tem/Trm) phenotypes, with upregulation of GZMA, GZMB, and GZMK and oligoclonal TCR expansion (42). Cui et al. characterized a highly clonally expanded CXCL13^+^ T-cell subset that, with CXCL9^+^ inflammatory monocytes, formed a pathogenic module centered on IFN-γ and CXCR3-ligand signaling (48), implicating sustained antigen presentation and local chemokine networks in perpetuating effector T-cell expansion.

In a multi-tissue analysis integrating lung tissue, BALF, and peripheral blood, Lin et al. demonstrated enrichment of CXCR6-expressing tissue-resident memory CD8⁺ T cells in CIP lung tissue, accompanied by elevated IFNG and STAT1 expression, enhanced TCR clonal expansion, and reduced repertoire diversity, with expanded clonotypes predominantly associated with Tem-GZMK and Trm populations (25). Although inhibitory-receptor gene-set activity did not differ significantly between CIP and control groups, this finding does not exclude exhaustion-associated states; computational analyses further suggested enhanced T-cell–myeloid interactions and predicted CXCL9/10/11–CXCR3-mediated recruitment of T cells by inflammatory macrophages (25).

Under prolonged antigen exposure, a subset of activated CD8^+^ T cells may enter an exhaustion program characterized by progressive loss of effector function, sustained co-inhibitory receptor expression, and stable epigenetic remodeling (54). Exhausted cells comprise a self-renewing progenitor-like subset (Tpex) and a terminally exhausted subset; PD-1 blockade preferentially expands Tpex, but persistent antigen ultimately drives terminal exhaustion (55). Because this framework was established under tumor neoantigen stimulation, the relevant antigens and kinetics in CIP remain uncharacterized. Zhou et al. reconstructed a pseudotime-derived activation-to-exhaustion continuum among alveolar ZNF683^hi tissue-resident CD8^+^ T cells in CIP (49), which, inferred from cross-sectional data, requires longitudinal validation. Sustained TCR–NFAT signaling induces TOX, which may stabilize these states through transcriptional and epigenetic reprogramming, although whether it impairs glucocorticoid responsiveness in CIP is unknown (56).

Corticosteroid-refractory CIP may therefore arise partly from clonal expansion of lung-resident CD8^+^ Trm cells driven by persistent antigenic stimulation (25, 42, 48) and from T cells distributed along an inferred activation-to-exhaustion continuum (54–56). Because glucocorticoids may incompletely eliminate established lung-resident populations, prospective longitudinal studies with TCR clonal tracking and treatment-response stratification are required.

#### 3.1.2 Th17/IL-17 inflammatory axis and glucocorticoid insensitivity

The Th17/IL-17 axis represents a second major adaptive pathway implicated in persistent lung inflammation in CIP. Wang et al. reported increased IL-17A in plasma and BALF at CIP onset, with increased Th17-cell frequency and an elevated Th17/Treg ratio that declined after clinical recovery (57), findings that support involvement of the axis without establishing it as specific to refractory disease.

Franken et al. identified in CIP BALF pathogenic Th17.1 cells co-expressing RORC, TBX21, IL17A, IFNG, and CSF2 (GM-CSF), a hybrid Th17/Th1 phenotype accompanied by inflammatory monocytes highly expressing IL1B, IL6, IL23A, and TNF; ligand–receptor analysis suggested that Th17.1-derived GM-CSF activates monocytes whose IL-23 sustains Th17.1 expansion in a feedback loop awaiting functional validation (50).

At the molecular level, IL-17A engagement of the IL-17RA/RC complex on lung epithelial cells, fibroblasts, and endothelial cells activates ACT1-dependent NF-κB and MAPK pathways and stabilizes inflammatory transcripts, amplifying neutrophilic and myeloid inflammation (58). Importantly, Th17-mediated neutrophilic inflammation is inherently less susceptible to glucocorticoid suppression: in murine models it was markedly resistant to dexamethasone, unlike glucocorticoid-sensitive Th2 eosinophilic inflammation (59); IL-17A directly impairs glucocorticoid responsiveness in human bronchial epithelial cells via PI3K signaling (60); and IL-17A can interact with glucocorticoids to induce aberrantly high CSF3 (G-CSF) expression, prolonging neutrophil survival (61). These insights originate from airway disease models and require validation in CIP, as does the altered GRα/GRβ balance implicated in glucocorticoid insensitivity in chronic inflammatory diseases (62).

Human CIP specimens provide direct evidence for involvement of the Th17/IL-17 axis, including increased IL-17A, expansion of Th17 cells, an increased Th17/Treg ratio, and enrichment of pathogenic Th17.1 cells accompanied by inflammatory monocytes (50, 57). However, the proposed link between this axis and glucocorticoid insensitivity remains largely inferential: steroid-resistant Th17-driven inflammation, IL-17A-mediated impairment of glucocorticoid responsiveness, aberrant CSF3 induction, and altered glucocorticoid receptor signaling have been demonstrated predominantly in experimental models or other inflammatory airway diseases rather than in corticosteroid-refractory CIP (59–62). Thus, although a Th17.1–myeloid circuit coupled with IL-17-associated glucocorticoid insensitivity provides a biologically plausible model of corticosteroid refractoriness, its causal relevance in CIP remains unproven and requires prospective comparison of corticosteroid-responsive and corticosteroid-refractory patient specimens.

### 3.2 Dysregulated inflammatory cytokine networks and JAK/STAT signaling

Corticosteroid-refractory CIP is unlikely to be driven by any single cytokine, but rather emerges from a self-amplifying network in which sustained T-cell activation, myeloid-cell reprogramming, and alveolar structural injury mutually reinforce one another. IL-6, TNF-α, and GM-CSF promote inflammatory-cell survival and differentiation, amplify tissue injury, and mediate T-cell–myeloid-cell crosstalk. IL-6 and GM-CSF primarily engage JAK/STAT-associated programs, whereas TNF-α signals through TNFR-dependent NF-κB, JNK, and p38 MAPK pathways, and crosstalk across these pathways may sustain inflammation despite partial glucocorticoid suppression. Because direct comparative data are lacking, the models presented here remain inferential.

#### 3.2.1 IL-6–JAK1/2–STAT3 signaling axis

IL-6 serves as a critical hub linking innate immunity, adaptive immunity, and pulmonary inflammation. Kowalski et al. reported significantly elevated IL-6 levels in BALF from patients with CIP (39), and Ploch et al. observed higher BAL IL-6 in CIP than in comparator groups, although only three patients with CIP were included (41). IL-6 elevation is not disease-specific and occurs in infection, acute respiratory distress syndrome, and drug-induced lung injury; however, single-cell analyses show that pro-inflammatory monocytes enriched in CIP BALF express high IL6, IL1B, TNF, and multiple chemokines and communicate extensively with activated T cells (48), suggesting that IL-6 contributes to local inflammatory amplification rather than serving as a disease-specific biomarker.

Mechanistically, IL-6 signals through membrane receptor-mediated pathways and soluble receptor complexes engaging gp130, activating JAK1/JAK2–STAT3, MAPK, and PI3K–AKT cascades (63). Sustained IL-6–STAT3 signaling promotes effector T-cell survival, cooperates with IL-1β and IL-23 in maintaining pathogenic Th17/Th17.1 responses, and enhances endothelial activation and myeloid recruitment. Tocilizumab has been used in small case series for steroid-refractory irAEs with limited CIP representation (35), and a patient with steroid-dependent CIP was weaned off corticosteroids after subcutaneous tocilizumab (20). IL-6 is therefore best regarded as one candidate amplifying pathway rather than an established determinant of refractoriness (64).

#### 3.2.2 TNF-α-mediated inflammatory amplification

TNF-α is produced predominantly by activated monocytes, macrophages, and effector T cells, positioning it at the interface of innate and adaptive immunity. Zhang et al. showed that clonally expanded effector memory and tissue-resident CD8^+^ T cells in CIP BALF are accompanied by elevated TNF-α, IFN-γ, and CXCL13 (42), and Cui et al. identified TNF^+^, IL-1β^+^, and IL-6^+^ pro-inflammatory monocytes within a pathogenic module providing mutual activation between T cells and myeloid cells (48).

Through TNFR1 or TNFR2, TNF-α activates NF-κB, JNK, and p38 MAPK signaling and promotes expression of adhesion molecules, chemokines, and pro-inflammatory cytokines; TNFR1 may additionally initiate necroptosis via the RIPK1/RIPK3/MLKL pathway, potentially amplifying alveolar barrier disruption, although direct evidence in CIP is lacking (65, 66). Infliximab has been used in steroid-refractory CIP, but Balaji et al. reported high rates of intensive care admission and mortality (67), and Beattie et al. found durable improvement in only a subset of patients, with substantial infectious risk (29). These outcomes may reflect severe baseline disease, selection bias, delayed escalation, and infection, leaving it unclear whether limited efficacy reflects inadequate pathway targeting or the multimechanistic nature of advanced disease.

#### 3.2.3 GM-CSF and T cell–myeloid cell bidirectional crosstalk

GM-CSF may constitute a critical molecular bridge linking pathogenic T cells to inflammatory myeloid cells. Franken et al. showed that Th17.1 cells in CIP BALF highly express CSF2, while co-enriched monocytes express CSF2RA and CSF2RB along with IL1B, IL6, IL23A, and TNF, and proposed a bidirectional loop in which Th17.1-derived GM-CSF activates monocytes whose IL-1β and IL-23 reciprocally sustain Th17.1 expansion (50).

RORγt-driven GM-CSF production by helper T cells is indispensable for T cell-mediated tissue inflammation in autoimmune neuroinflammation models (68), although its contribution in CIP requires direct investigation. Beyond hematopoiesis, GM-CSF promotes the survival, differentiation, and antigen-presenting capacity of myeloid cells and augments their production of TNF-α, IL-1β, and IL-6, and sustained signaling could plausibly maintain a GM-CSF–myeloid–IL-1β/IL-6/IL-23 amplification circuit (69, 70).

#### 3.2.4 Convergence of IFN-γ-, IL-6-, IL-23-, and GM-CSF-associated JAK/STAT signaling in steroid refractoriness

The cytokine axes described above—IFN-γ from CD8^+^ Trm cells, IL-6 and IL-23 from inflammatory myeloid cells, and GM-CSF from Th17.1 cells—activate distinct but interconnected branches of the JAK/STAT network. IFN-γ activates JAK1/JAK2–STAT1 signaling, promoting CXCL9/10/11 expression and antigen presentation; IL-6 predominantly engages JAK1-associated STAT3, IL-23 signals through JAK2/TYK2–STAT3 to support Th17/Th17.1 responses, and GM-CSF mainly activates JAK2–STAT5 while additionally engaging MAPK and PI3K pathways (71–73). TNF-α, by contrast, activates TNFR-dependent NF-κB, JNK, and p38 MAPK pathways (65, 66). Crosstalk among these systems may sustain chemokine production, immune-cell recruitment, and reciprocal T-cell–myeloid-cell activation.

This convergent architecture may contribute to incomplete glucocorticoid responsiveness through redundant mechanisms: sustained STAT1/STAT3 signaling may maintain inflammatory and survival programs, persistent lung-resident Trm cells may re-establish IFN-γ–STAT1 signaling during tapering, and inhibition of a single upstream mediator may leave compensatory signaling through parallel pathways.

Cross-organ irAE research provides translational support for JAK inhibition. Sasson et al. identified IFN-γ-producing CD8^+^ Trm cells as the dominant effector population in ICI-related colitis and obtained proof-of-concept evidence for tofacitinib in a refractory case, a phenotype analogous to the IFN-γ–STAT1 network in CIP, although extrapolation across tissues is not warranted (74). In a prospective cohort of 29 patients with severe or refractory irAEs, only two of whom had CIP, Habib et al. reported potential benefit in a subset (75).

IL-6, TNF-α, and GM-CSF are unlikely to act as independent effectors; they participate in an interconnected network mediated by JAK/STAT-, NF-κB-, and MAPK-associated signaling in which crosstalk and partial redundancy may allow inflammatory programs to persist despite suppression of individual mediators: GM-CSF sustains inflammatory myeloid-cell survival and activation (50, 69, 70), TNF-α promotes immune-cell recruitment and tissue injury (42, 48, 65, 66), and IL-6–STAT3 signaling supports effector T-cell survival and Th17-related inflammation (39, 41, 63). This multibranch architecture provides a plausible explanation for the inconsistent efficacy of sequential single-cytokine blockade (29, 67).

### 3.3 Innate immune dysregulation

Although CIP has traditionally been conceptualized as T cell-mediated, single-cell analyses indicate substantial innate immune remodeling, including inflammatory myeloid expansion, aberrant neutrophil recruitment, and enhanced antigen-presenting cell function. These populations may modulate adaptive activation through IL-6, TNF-α, GM-CSF, and CXCL chemokines, establishing bidirectional adaptive–innate circuits that may contribute to insufficient glucocorticoid responsiveness.

#### 3.3.1 Alveolar macrophages as central orchestrators of persistent inflammation

Suresh et al. provided early evidence that the alveolar immune landscape is profoundly altered in CIP, with expansion of a CD11b-high inflammatory myeloid compartment and of central memory T cells, and impaired regulatory T-cell function (76). Cui et al. characterized an expanded CXCL9^+^ pro-inflammatory monocyte/macrophage population in CIP BALF highly expressing IL1B, IL6, TNF, and chemokines, forming a tightly interactive module with CXCL13^+^ activated T cells through the CXCL9/10/11–CXCR3 axis (48), and Lin et al. found CIP lung macrophages to exhibit an enhanced phagocytic, IL-1β-high phenotype coupled to CD8^+^ Trm cells (25).

Mechanistically, sustained ICI-driven lymphokine stimulation may skew alveolar macrophages toward a persistent pro-inflammatory state, maintaining TNF-α, IL-1β, and IL-6 production while impairing acquisition of resolving and reparative programs (77, 78). Macrophage activation states *in vivo* form a continuum rather than a fixed M1/M2 dichotomy, and CIP-relevant subsets require further definition (79). Franken et al. additionally showed that IL1B^+^IL6^+^ monocytes form reciprocal loops with CSF2hi Th17.1 cells (50), whereas whether ICI-driven stimulation induces a trained immunity-like state in alveolar macrophages remains speculative (80).

#### 3.3.2 Gasdermin-mediated pyroptosis and inflammasome-associated tissue injury

Pyroptosis and gasdermin-mediated inflammatory cell death may provide an additional mechanistic link between innate immune activation and tissue injury in CIP. Unlike apoptosis, pyroptosis is a lytic and proinflammatory form of regulated cell death characterized by gasdermin-mediated membrane pore formation, loss of membrane integrity, and release of intracellular inflammatory mediators and damage-associated molecular patterns (81, 82). Recent single-cell profiling of lung tissue from patients with CIP has provided CIP-specific evidence implicating this pathway. Lin et al. identified enrichment of pyroptosis-related transcriptional programs in CIP-associated macrophages, with increased expression of GSDME, particularly in tissue-resident macrophage populations. Increased GSDME expression was further supported by bulk transcriptomic and immunofluorescence analyses, while an increased proportion of cleaved N-terminal GSDME (GSDME-N)-positive macrophages was detected in BALF and circulating GSDME concentrations were higher in patients with CIP. These findings suggest that GSDME-mediated macrophage pyroptosis may contribute to pulmonary immune injury in CIP (25).

Mechanistically, gasdermin cleavage generates pore-forming N-terminal fragments that disrupt the plasma membrane, converting intracellular inflammatory signaling into lytic cellular injury and release of proinflammatory contents (81, 82). This process may establish a feed-forward circuit in which activated or pyroptotic macrophages amplify local cytokine signaling, recruit additional immune cells, and propagate alveolar and interstitial injury. Inflammasome-associated signaling may further intersect with this process, as increased IL-1β has been observed in CIP-associated myeloid cells and inflammasome-related pathways are enriched in CIP tissue (25). Thus, pyroptosis provides a plausible mechanistic bridge between myeloid-cell activation and the acute parenchymal damage observed in severe CIP. However, current evidence does not establish whether GSDME activation is a driver of corticosteroid refractoriness, a marker of severe tissue inflammation, or a downstream consequence of lung injury. Moreover, the relative contributions of canonical inflammasome–caspase-1–GSDMD signaling and alternative GSDME-dependent pyroptosis remain incompletely defined in CIP (25, 81, 82). These pathways should therefore be considered emerging mechanistic candidates rather than validated therapeutic targets.

#### 3.3.3 Potential contribution of neutrophils and neutrophil extracellular traps

Neutrophil extracellular traps (NETs) are chromatin networks decorated with histones, myeloperoxidase (MPO), and neutrophil elastase released by activated neutrophils, and excessive formation can drive tissue injury and immune amplification (83). Zheng et al. identified aberrant basaloid epithelial cells enriched in severe CIP with increased SOX9 and CXCL3/CXCL5 expression, suggesting CXCR2-mediated neutrophil recruitment (84), and Lin et al. observed enhanced granulocyte-recruitment chemokine networks in CIP lung tissue (25). NET-associated histones, MPO, and reactive oxygen species disrupt the alveolar–capillary barrier and stimulate macrophage cytokine production, and excessive NET accumulation has been associated with epithelial injury across respiratory diseases (85), including NET-driven injury in COVID-19-associated ARDS (86) and in airway inflammatory disease (87). Because these findings originate from non-CIP settings, neutrophilic infiltration should not be equated with NET formation in CIP.

#### 3.3.4 Dendritic cells and sustained antigen presentation

Cui et al. identified LAMP3^+^ dendritic cells (DCs) in CIP BALF that appear to derive from CXCL9^+^ monocytes and interact with effector T cells through CXCL9/10/11–CXCR3 and IFNG–IFNGR signaling (48), and Lin et al. documented enhanced antigen-presentation programs associated with the IFN-γ milieu (25). Following ICI-mediated relief of PD-1/PD-L1 inhibition, activated DCs may enhance co-stimulation and contribute to local antigen presentation—potentially involving tumor-derived, microbial, cross-reactive, or pulmonary self-antigens, none definitively identified in CIP (88).

Innate immune dysregulation in CIP thus manifests as coordinated multilineage dysfunction in which pro-inflammatory monocytes/macrophages, gasdermin-mediated inflammatory cell death, neutrophil-associated injury, and dendritic-cell antigen presentation may establish mutually reinforcing adaptive–innate circuits. Pyroptotic macrophage death may provide an additional mechanism through which immune activation is translated into membrane disruption, release of inflammatory cellular contents, and propagation of parenchymal injury. However, the relative contribution of these processes to corticosteroid refractoriness remains uncertain. Persistent pyroptotic and cytokine-mediated injury may, in turn, increase the burden of epithelial damage that must be resolved through subsequent reparative programs, providing a potential link between acute innate immune injury and the maladaptive remodeling discussed below.

### 3.4 Aberrant tissue repair and pulmonary remodeling

Steroid-refractory CIP may reflect not only failure of inflammation control but also a transition from reversible immune-mediated injury toward aberrant wound healing and structural remodeling, involving persistent alveolar epithelial injury, impaired type 2 alveolar epithelial cell (AT2) regeneration, accumulation of aberrant transitional epithelial cells, retention of monocyte-derived macrophages, and TGF-β-driven fibroblast activation. Aberrant repair may promote steroid resistance, follow from persistent inflammation, or both.

#### 3.4.1 Persistent alveolar injury and dysregulated repair programs

Naidoo et al. defined chronic CIP as pneumonitis that persists or worsens during tapering after ICI discontinuation and requires immunosuppression for at least 12 weeks, with organizing pneumonia, intra-alveolar fibrin organization, and persistent Ki67^+^PD-1^+^ lymphocytes indicating failure to enter a resolution phase (23). Complementing these observations, Zhou et al. described a temporal evolution of unselected CIP from an acute inflammation-dominant phase toward subacute organization and, in some patients, chronic fibrotic remodeling after approximately 13 weeks (24). Histologically, acute fibrinous pneumonitis predominated early, organizing pneumonia became more prominent during the subacute phase, and the limited chronic-phase tissue available showed fibrosis and disruption of normal lung architecture. These findings support the concept of an inflammation-to-remodeling continuum in CIP, although whether corticosteroid-refractory disease preferentially follows this trajectory remains unknown. Imran et al. characterized two major patterns: severe cases predominantly exhibiting acute lung injury (ALI)/diffuse alveolar damage (DAD) with rapid progression, and less severe cases exhibiting organizing pneumonia or chronic interstitial inflammation (89); a small series of 12 patients identified alveolitis, organizing features, and focal fibrosis (90).

Normal alveolar repair requires AT2 proliferation and differentiation into type 1 alveolar epithelial cells (AT1); sustained injury may arrest this process, promoting local coagulation, fibrin deposition, and fibroblast activation (91). In CIP, activated CD8^+^ Trm cells and inflammatory myeloid populations may expose the epithelium to a sustained milieu of IFN-γ, TNF-α, IL-1β, reactive oxygen species, and proteases, and single-cell studies have documented enhanced immune–epithelial interactions (25) and aberrant SOX9-upregulating basal-like epithelial cells recruiting neutrophils via CXCL3/CXCL5 in severe CIP (84).

#### 3.4.2 AT2 cell transitional state and regenerative arrest

Following lung injury, AT2 cells pass through a transient injury-associated progenitor state rather than differentiating directly into AT1 cells. Choi et al. showed that macrophage-derived IL-1β induces IL1R1-expressing AT2 cells to become damage-associated transient progenitors via HIF1α-mediated glycolytic reprogramming, and that sustained IL-1β exposure prevents completion of AT1 differentiation, causing pathological accumulation and impaired regeneration (51)—a mechanism relevant to CIP, in which pro-inflammatory myeloid cells may provide a sustained IL-1β source. KRT8^+^ transitional and PATS (pre-alveolar type-1 transitional cell state) cells are transient during normal repair but persist aberrantly in fibrosis, enriched for TP53, TGF-β, DNA damage response, and senescence signatures (92, 93). Sustained inflammation may therefore promote repeated entry into, or delayed exit from, these states, converting epithelial cells into sources of pro-fibrotic signals within a self-amplifying loop involving profibrotic macrophages and myofibroblasts (94) that is prone to senescence (95).

#### 3.4.3 Macrophage–TGF-β–fibroblast axis and structural remodeling

Misharin et al. demonstrated in a bleomycin-induced murine fibrosis model that monocyte-derived alveolar macrophages recruited after injury persist long-term and that their selective depletion significantly attenuated fibrosis, whereas tissue-resident alveolar macrophages did not contribute (52). The inflammatory myeloid accumulation observed in CIP may therefore sustain a pro-fibrotic niche even after partial glucocorticoid suppression of T cell-driven inflammation, with activated macrophages releasing TGF-β, platelet-derived growth factor, chemokines, and matrix-regulatory molecules that drive myofibroblast differentiation (78).

TGF-β is the central mediator of the inflammation-to-fibrosis transition, promoting expression of α-smooth muscle actin, COL1A1, and fibronectin through SMAD2/3 and non-canonical MAPK pathways while inhibiting epithelial repair and enhancing myofibroblast survival (96). In single-cell atlases of human pulmonary fibrosis, aberrant basal-like and transitional epithelial cells co-localize with pro-fibrotic mesenchymal cells in remodeling niches maintained by TGF-β, integrin, and matrix signaling (97), and as CIP progresses toward an organizing or fibrotic phase the dominant drivers may shift from immune infiltration to a self-sustaining epithelial–macrophage–fibroblast network.

Aberrant tissue repair may contribute to glucocorticoid hyporesponsiveness in three ways: glucocorticoids cannot restore arrested AT2–AT1 differentiation; monocyte-derived macrophages and myofibroblasts persist and maintain TGF-β and matrix production despite partial suppression of inflammation; and once matrix deposition and architectural disruption are established, radiological and physiological abnormalities may become partly uncoupled from active inflammation. Clinically, incomplete radiological resolution has been associated with persistent symptoms (17). This supports a provisional distinction between a persistent inflammation subtype and an aberrant repair/structural remodeling subtype for which regenerative or anti-fibrotic approaches may be more appropriate.

### 3.5 Proposed mechanistic framework of corticosteroid refractoriness in CIP

Based on currently available indirect and translational evidence, we propose four non-mutually exclusive mechanisms. These mechanisms are supported by different levels of evidence, ranging from direct experimental evidence of glucocorticoid insensitivity in airway structural cells to CIP-specific single-cell associations and cross-disease mechanistic extrapolation. The mechanistic categories reviewed above converge on a shared phenotypic outcome—persistent pulmonary inflammation that resists glucocorticoid suppression—through distinct and partially independent processes that may co-exist within the same patient (**Figure 1**). Recognizing their co-existence is clinically important because it implies that no single pharmacological intervention is likely to be universally effective, and that response to rescue therapy may depend critically on which mechanism predominates in an individual patient at a given disease stage.

**Figure 1:**
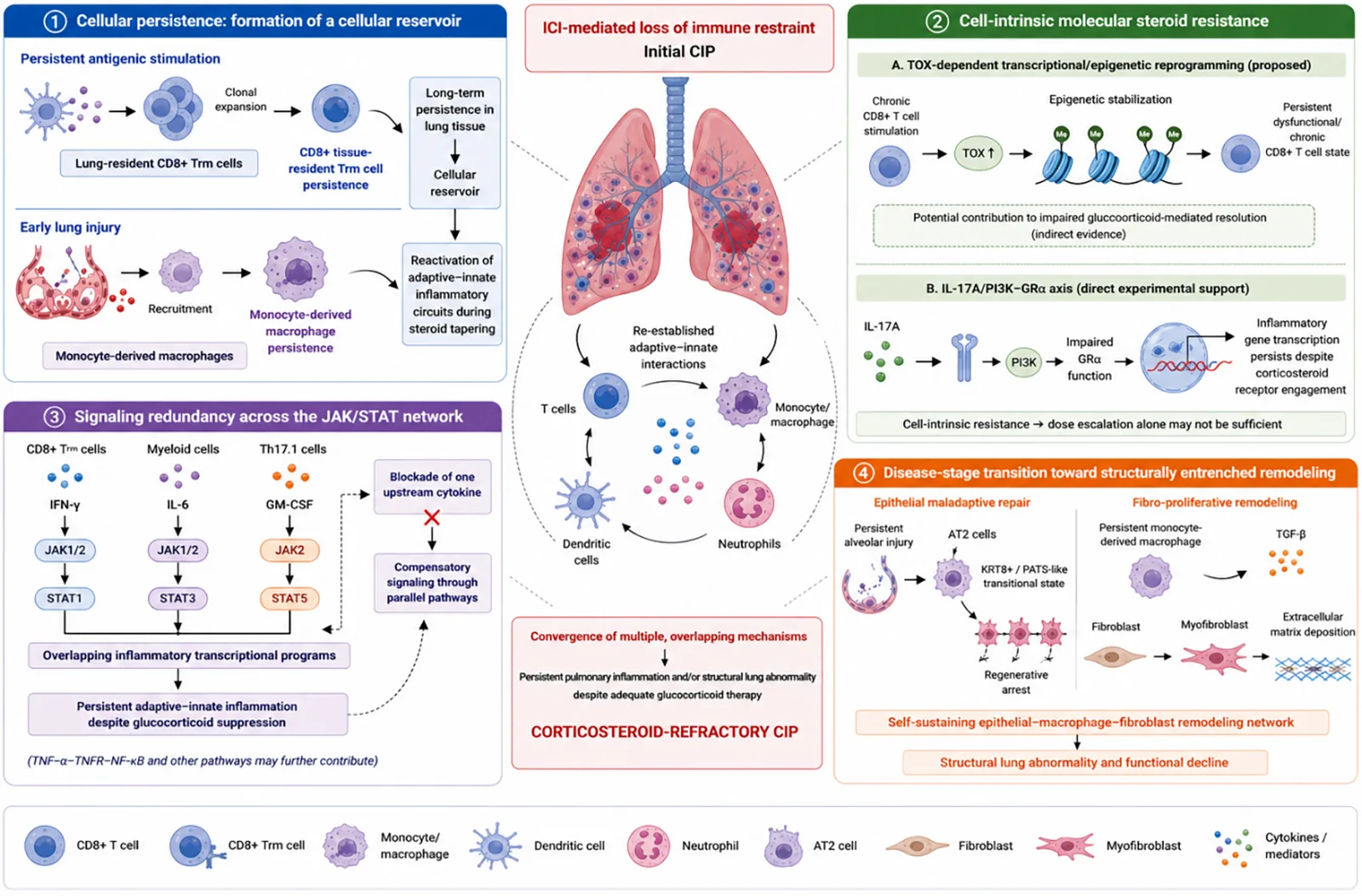
Proposed mechanistic framework of corticosteroid refractoriness in checkpoint inhibitor pneumonitis. Corticosteroid-refractory checkpoint inhibitor pneumonitis (CIP) is proposed to arise from four non-mutually exclusive and potentially overlapping mechanisms (1). Cellular persistence: clonally expanded lung-resident CD8^+^ tissue-resident memory (Trm) cells and recruited monocyte-derived macrophages may persist within the lung and form a cellular reservoir capable of re-establishing adaptive–innate inflammatory circuits during glucocorticoid tapering (2). Cell-intrinsic molecular steroid resistance: TOX-dependent transcriptional and epigenetic reprogramming may stabilize chronically stimulated CD8^+^ T-cell states, although its contribution to glucocorticoid refractoriness in CIP remains unproven. In parallel, IL-17A–PI3K signaling may impair glucocorticoid receptor-α (GRα) function in structural lung cells, allowing inflammatory gene transcription to persist despite corticosteroid receptor engagement (3). Signaling redundancy across the JAK/STAT network: IFN-γ–STAT1, IL-6–STAT3, and GM-CSF–STAT5 pathways provide partially overlapping inflammatory signals from distinct cellular sources. Consequently, suppression of a single upstream mediator may permit compensatory signaling through parallel pathways and sustain adaptive–innate inflammation (4). Disease-stage transition toward structurally entrenched remodeling: persistent alveolar injury may promote accumulation of KRT8^+^/PATS-like transitional epithelial states and regenerative arrest, while persistent monocyte-derived macrophages, TGF-β signaling, myofibroblast activation, and extracellular matrix deposition may establish a self-sustaining epithelial–macrophage–fibroblast remodeling network. As disease evolves, structural abnormalities may become partly uncoupled from active immune inflammation and therefore less responsive to further immunosuppression. These mechanisms may coexist and shift in relative dominance over time, supporting longitudinal mechanistic stratification rather than a uniform approach to rescue therapy. AT2, alveolar type 2 epithelial cell; CIP, checkpoint inhibitor pneumonitis; CD8^+^ Trm, CD8^+^ tissue-resident memory T cell; ECM, extracellular matrix; GM-CSF, granulocyte–macrophage colony-stimulating factor; GRα, glucocorticoid receptor alpha; HRCT, high-resolution computed tomography; IFN-γ, interferon gamma; IL, interleukin; JAK, Janus kinase; PATS, pre-alveolar type-1 transitional state; PI3K, phosphoinositide 3-kinase; STAT, signal transducer and activator of transcription; TCR, T-cell receptor; TGF-β, transforming growth factor beta; TNF-α, tumor necrosis factor alpha; TOX, thymocyte selection-associated high mobility group box.

First, cellular persistence. Lung-resident CD8^+^ Trm cells generated through persistent antigenic stimulation and clonal expansion (25, 42, 48), together with monocyte-derived macrophages recruited during early lung injury (52), may exhibit long-term persistence within the lung parenchyma. Compared with circulating inflammatory cells, tissue-resident immune populations may be less susceptible to depletion and may persist after partial clinical improvement, although direct evidence supporting this mechanism in steroid-refractory CIP remains limited. Persistent tissue-resident immune populations could provide a cellular reservoir capable of reactivating inflammatory programs during glucocorticoid tapering, although longitudinal clonal tracking is required to confirm this possibility. Such persistent immune populations may contribute to recurrent inflammation by re-establishing adaptive–innate immune interactions described in Sections 3.1 and 3.3.

Second, molecular steroid resistance at the cellular level. Two intracellular processes may limit durable inflammatory resolution, although only IL-17-associated impairment of glucocorticoid responsiveness has direct experimental support in airway structural cells. TOX-dependent transcriptional and epigenetic reprogramming can stabilize exhausted or chronically stimulated CD8^+^ T-cell differentiation states (56). Whether this epigenetic stability limits glucocorticoid-mediated resolution in CIP remains unknown. Independently, IL-17A signaling through PI3K reduces glucocorticoid responsiveness via decreased HDAC2 activity in human bronchial epithelial cells, sustaining inflammatory gene expression despite corticosteroid treatment (60). These mechanisms are cell-intrinsic and are therefore not addressed by dose escalation alone.

Third, signaling redundancy across the JAK/STAT network. The JAK/STAT signaling network receives convergent inputs from IFN-γ–STAT1, IL-6–STAT3, and GM-CSF–STAT5 pathways arising from distinct cellular sources (71–73). Because these cytokines engage partially overlapping JAK-family kinases and convergent but non-identical STAT-dependent programs, suppression of a single upstream cytokine may leave compensatory signaling through parallel pathways. This architectural redundancy may allow inflammatory signaling to persist despite substantial glucocorticoid-mediated suppression of individual transcriptional programs, thereby sustaining adaptive–innate inflammatory interactions. Such signaling redundancy may represent one possible explanation for the limited and inconsistent efficacy reported with sequential single-cytokine blockade (29, 67), although additional factors, including disease severity, treatment timing, and biological heterogeneity, are also likely to contribute.

Fourth, disease-stage transition toward structurally entrenched remodeling. We hypothesize that, in some patients, progression from an inflammatory to an organizing or fibrotic phase may involve a shift from cytokine-dominant alveolitis toward a self-sustaining epithelial–macrophag–fibroblast remodeling network (52, 78, 96, 97). Once AT2 cells are arrested in aberrant KRT8^+^/PATS-like transitional states (51, 92–94) and myofibroblasts have established extracellular matrix deposition, radiological and functional abnormalities may become partly uncoupled from ongoing immune inflammation. Whether an analogous transition contributes to corticosteroid refractoriness in CIP remains unproven. If such a transition occurs, further escalation of immunosuppression may have diminishing biological justification while increasing treatment-related risks, a hypothesis that requires prospective validation in CIP.

Taken together, these four resistance mechanisms indicate that steroid-refractory CIP is not a homogeneous clinical entity but a biologically heterogeneous syndrome in which the dominant barrier to treatment response differs between patients and may shift over time within the same patient. This heterogeneity carries direct clinical implications: effective rescue therapy is unlikely to follow a single algorithmic protocol, but will instead require mechanistic stratification—guided by longitudinal BALF immunophenotyping, cytokine profiling, TCR clonal dynamics, and serial HRCT—to distinguish patients whose refractory course is driven by persistent cellular inflammation (potentially responsive to JAK inhibition or targeted cytokine blockade) from those whose disease has transitioned to structurally entrenched remodeling (for whom anti-fibrotic or regenerative strategies may be more appropriate). Prospective studies that stratify patients according to these mechanistic subtypes, rather than treating steroid-refractory CIP as a single clinical category, represent the most critical unmet need in this field.

Despite the widespread clinical use of corticosteroids, canonical mechanisms of glucocorticoid resistance—including altered GRα/GRβ balance, impaired glucocorticoid receptor nuclear translocation, HDAC2 dysfunction, persistent AP-1/NF-κB activation, and oxidative stress-mediated receptor impairment—have been extensively characterized in chronic inflammatory diseases but have not yet been systematically investigated in CIP (62, 98). Whether these classical molecular pathways contribute to steroid-refractory CIP remains unknown and represents an important priority for future translational research.

## 4 Current treatment strategies

### 4.1 Appropriate use of glucocorticoids

Glucocorticoids are the first-line treatment for CIP, and their appropriate administration is a prerequisite for any escalation to second-line immunosuppression. For grade 2 CIP, ICI therapy should be withheld and systemic glucocorticoids initiated, most commonly prednisone or an equivalent at 1–2 mg/kg/day; once symptoms and imaging improve to grade 1 or below, resumption may be considered after risk–benefit assessment, whereas absence of improvement after 48–72 hours should prompt management as grade 3 disease. For grade 3–4 CIP, permanent ICI discontinuation is generally recommended with intravenous methylprednisolone or an equivalent at 1–2 mg/kg/day and empiric antibiotics as appropriate (10–12).

Following a favorable response, the dose should be tapered gradually according to symptoms, oxygenation, and radiological improvement, generally over no less than 4–6 weeks to reduce relapse. Because prolonged high-dose therapy increases the risk of serious infection, osteoporosis, and hyperglycemia, individualized tapering should begin once inflammation is controlled. The NCCN and ESMO guidelines recommend considering prophylaxis against Pneumocystis jirovecii pneumonia for prolonged moderate- to high-dose therapy, with the NCCN specifying a prednisone equivalent of ≥20 mg/day for ≥4 weeks and trimethoprim–sulfamethoxazole as preferred (10–12).

For grade 3–4 CIP that does not improve after 48 hours, escalation should be considered, including pulse corticosteroid therapy and second-line immunosuppressive agents. Pulse therapy, defined as doses exceeding 250 mg of prednisone or equivalent, has established efficacy in life-threatening autoimmune disease, but CIP evidence is confined to case reports: Lai K.C. et al. described two patients with grade 4 CIP who improved rapidly after methylprednisolone 500 mg for 3 days (9, 13). Decisions on second-line agents should account for disease status, infection risk, and multidisciplinary assessment (10, 11).

### 4.2 Guideline-endorsed rescue immunomodulation

No standard second-line regimen has been established for corticosteroid-refractory CIP. Across ASCO, ESMO, and NCCN guidance, suggested rescue options include mycophenolate mofetil (MMF), intravenous immunoglobulin (IVIG), infliximab, tocilizumab, and cyclophosphamide (10–12). These agents are summarized in **Table 1** and reviewed below.

**Table 1:** Evidence summary of second-line and rescue therapies for corticosteroid-refractory checkpoint inhibitor pneumonitis.

Agent or strategy	Mechanistic rationale	Evidence in corticosteroid-refractory CIP	Potential clinical role	Key safety considerations and implications when infection is uncertain
Pulse corticosteroid intensification	Rapid broad suppression of inflammatory transcriptional programs and immune-cell activation.	Only isolated case reports suggest possible benefit in severe CIP poorly responsive to conventional glucocorticoids; no prospective evidence defines dose, timing, or target population.	Early rescue consideration in selected severe cases before or alongside second-line immunomodulation; not a substitute for infection evaluation or supportive care.	May increase infectious risk, hyperglycemia, myopathy, delirium, and masking of infection; prolonged escalation may be ineffective if structural injury predominates.
Mycophenolate mofetil (MMF)	Inhibits inosine monophosphate dehydrogenase and suppresses *de novo* purine synthesis, preferentially limiting activated T- and B-cell proliferation.	Case reports and small retrospective cohorts; some patients improved, but independent efficacy is difficult to assess because of small sample sizes, heterogeneous disease, and combination regimens.	Guideline-listed option for non-fulminant or lymphocyte-dominant inflammatory phenotypes; may help steroid-dependent or taper-relapsing disease.	Infection, cytopenias, gastrointestinal toxicity, and hepatotoxicity; relatively slow onset limits its role as sole rescue therapy in fulminant disease. Use cautiously when active infection remains possible.
Infliximab	Neutralizes soluble and transmembrane TNF-alpha, reducing inflammatory-cell recruitment and macrophage-associated inflammatory amplification.	Retrospective cohorts and case reports show inconsistent outcomes; high mortality in some steroid-refractory cohorts likely reflects severe baseline disease, indication bias, delayed escalation, infection, or irreversible lung injury.	Possible salvage option for selected patients with persistent inflammatory phenotype after infection is excluded.	Substantial concern for serious bacterial, fungal, and mycobacterial infection; screen for tuberculosis and hepatitis B when feasible. Avoid in uncontrolled infection or sepsis.
Tocilizumab	Blocks IL-6 receptor signaling, attenuating IL-6-mediated inflammatory amplification and Th17-associated immune responses.	Evidence mainly from multi-organ irAE cohorts and case reports with limited CIP-specific data; no validated IL-6 threshold predicts response.	Potential option for selected cytokine-dominant or steroid-dependent/refractory phenotypes, but target population and timing remain undefined.	Bacterial, fungal, viral, and mycobacterial infection; neutropenia, thrombocytopenia, liver enzyme elevation, and gastrointestinal perforation. Rapid CRP suppression may mask ongoing infection and complicate monitoring.
Intravenous immunoglobulin (IVIG)	Broad immunomodulation through Fc receptor competition, modulation of dendritic cells and macrophages, complement inhibition, Treg promotion, and autoantibody neutralization.	Used in case reports, small series, and retrospective cohorts; independent efficacy remains uncertain.	Guideline-listed rescue option; may be considered when broader immunosuppression is undesirable or infection risk is high, and in fulminant disease as part of selected salvage regimens.	Does not cause the same degree of cellular immunosuppression and may be attractive when infection risk is high or broader immunosuppression is undesirable; nevertheless, thrombosis, renal dysfunction, hemolysis, infusion reactions, and volume overload remain relevant.
Cyclophosphamide	Alkylating cytotoxic immunosuppressant that suppresses rapidly proliferating T and B lymphocytes; extrapolated from severe autoimmune lung disease and vasculitis.	CIP evidence is limited to case reports and small studies; response is often reported within combination therapy, preventing assessment of independent benefit.	Exceptional rescue option for critically ill patients with rapidly progressive disease after other strategies fail or are unsuitable.	Profound immunosuppression, myelosuppression, opportunistic infection, hemorrhagic cystitis, and organ toxicity; reserve for exceptional rescue situations after careful exclusion of infection.
Calcineurin inhibitors (tacrolimus, cyclosporine)	Block calcineurin-NFAT signaling and reduce IL-2-dependent T-cell activation and proliferation.	Tacrolimus and cyclosporine experience is limited to case reports and small series; no standardized dosing, trough targets, or prospective data exist for CIP.	Exploratory salvage option for suspected T-cell-dominant or taper-relapsing disease in highly selected patients.	Opportunistic infection, nephrotoxicity, hypertension, neurotoxicity, metabolic effects, and major drug interactions; therapeutic drug monitoring is required.
Rituximab	CD20-directed B-cell depletion; theoretical rationale only if B-cell or autoantibody-mediated features are suspected.	Very limited evidence in severe or refractory CIP cohorts; available studies do not separately report rituximab-specific outcomes.	Not routine. Consider only in exceptional cases with autoimmune features or suspected B-cell-mediated mechanisms after multidisciplinary review.	Prolonged B-cell depletion, hypogammaglobulinemia, infection, hepatitis B reactivation, and impaired vaccine responses.
JAK inhibitors	Target convergent cytokine signaling downstream of IFN-gamma, IL-6, IL-23, and GM-CSF pathways.	Evidence in CIP is indirect or minimal; irAE cohorts include very few pneumonitis cases and do not establish CIP-specific efficacy, dosing, or timing.	Mechanism-directed experimental option for selected life-threatening or refractory irAEs; not established for corticosteroid-refractory CIP.	Serious infection, herpes zoster, myelosuppression, thromboembolism, cardiovascular risk, and uncertain effect on interferon-dependent antitumor immunity.
BTK inhibitors	May modulate B-cell receptor signaling and myeloid inflammatory pathways including NF-kB and inflammasome-associated responses.	No reliable clinical evidence demonstrates efficacy in corticosteroid-refractory CIP; support is preclinical or extrapolated from non-CIP inflammatory models.	Research direction rather than current rescue therapy; possible future relevance for myeloid- or B-cell-associated endotypes.	Infection, bleeding, atrial fibrillation, cytopenias, drug interactions, and uncertain antitumor immunological effects; target phenotype unvalidated.
IL-17 pathway inhibitors	IL-17/Th17 signaling promotes neutrophilic inflammation and has mechanistic links to glucocorticoid insensitivity in airway models.	No case series, cohort, or prospective study has evaluated IL-17 blockade in corticosteroid-refractory CIP. Evidence comes from CIP biomarker studies, other IL-17 diseases, and non-pulmonary irAEs.	Hypothesis-generating option only; future studies may evaluate Th17/IL-17-high phenotypes.	Risk of infection, especially mucocutaneous candidiasis; uncertain pulmonary safety and antitumor consequences.
Abatacept or anti-thymocyte globulin	Abatacept restores CTLA-4-mediated T-cell co-inhibitory signaling; anti-thymocyte globulin rapidly depletes T lymphocytes.	Abatacept evidence is mainly from severe immune-related myocarditis; anti-thymocyte globulin is limited to a single steroid-refractory pneumonitis case report.	Exceptional last-resort or highly selected T-cell-driven toxicity; not standard rescue therapy for CIP.	Profound immunosuppression, severe infection, cytopenias, serum sickness or infusion reactions, and possible attenuation of antitumor immunity.
Antifibrotic agents (nintedanib, pirfenidone)	Target fibroblast activation, growth factor signaling, TGF-beta-associated fibrosis, and extracellular matrix deposition.	CIP evidence is limited to case reports and retrospective observations; no prospective data define efficacy or patient selection.	Investigational adjunct in selected patients with progressive fibrotic change after active inflammation is controlled; should not replace immunosuppression when inflammatory alveolitis is ongoing.	Gastrointestinal toxicity, liver enzyme elevation, bleeding risk with nintedanib, photosensitivity with pirfenidone; optimal timing in CIP unknown.
Combination or sequential second-line immunosuppression	Attempts to target overlapping inflammatory mechanisms when monotherapy fails; examples include IVIG plus infliximab, tacrolimus plus cyclophosphamide, MMF followed by infliximab, or IVIG plus MMF.	Evidence remains anecdotal and heterogeneous. Some cases improved, whereas IVIG plus infliximab in a critically ill cohort was associated with poor outcomes, likely confounded by disease severity and infection.	Individualized salvage strategy for progressive disease despite glucocorticoids and one immunomodulator; no specific dual-agent regimen is standard.	High infection risk, organ toxicity, difficulty attributing response, publication bias, and confounding by indication. Requires repeated exclusion of infectious mimics.

This table summarizes reported and proposed rescue strategies rather than recommending a fixed treatment hierarchy. Evidence levels are generally low because most data derive from case reports, small retrospective cohorts, multi-organ irAE cohorts, or mechanistic extrapolation. Treatment selection should be individualized after exclusion of infection, tumor progression, and other mimics.

BALF, bronchoalveolar lavage fluid; CIP, checkpoint inhibitor pneumonitis; CTLA-4, cytotoxic T-lymphocyte-associated antigen 4; ECM, extracellular matrix; GM-CSF, granulocyte-macrophage colony-stimulating factor; ICI, immune checkpoint inhibitor; IL, interleukin; irAE, immune-related adverse event; IVIG, intravenous immunoglobulin; JAK, Janus kinase; MMF, mycophenolate mofetil; NETs, neutrophil extracellular traps; NFAT, nuclear factor of activated T cells; NF-kB, nuclear factor kappa B; PD-1, programmed death-1; PD-L1, programmed death-ligand 1; STAT, signal transducer and activator of transcription; TGF-beta, transforming growth factor-beta; TNF-alpha, tumor necrosis factor-alpha.

#### 4.2.1 Mycophenolate mofetil

MMF is rapidly converted *in vivo* to mycophenolic acid, which selectively inhibits inosine monophosphate dehydrogenase and reduces *de novo* guanosine nucleotide synthesis; because activated T and B lymphocytes depend disproportionately on this pathway, MMF relatively selectively suppresses lymphocyte proliferation (99). ASCO lists MMF among the agents that may be considered for corticosteroid-refractory CIP (11), although direct evidence derives from case reports, small series, and retrospective studies (29, 100). Shioiri et al. reported two patients with corticosteroid-resistant CIP in whom symptoms, oxygenation, and radiological findings improved after adding MMF, with both agents ultimately discontinued without recurrence (100), whereas a retrospective study of 26 patients included six treated with MMF-containing regimens whose independent efficacy could not be assessed (29). MMF may therefore benefit some patients with refractory, dependent, or relapsing disease, although its optimal use remains undefined. Given its relatively delayed onset of immunosuppressive effect, MMF may be less suitable as sole emergency salvage therapy in rapidly progressive severe CIP (11, 15).

#### 4.2.2 Infliximab

Infliximab is a chimeric IgG1 monoclonal antibody that neutralizes soluble and transmembrane tumor necrosis factor-α (TNF-α), inhibiting inflammatory cell recruitment, macrophage activation, and amplification of the inflammatory cascade, and is listed in several guidelines as an option for escalated immunomodulation (11). Supporting evidence remains limited and variable: Balaji et al. found persistently high treatment failure and mortality among patients receiving infliximab-containing immunomodulation (67), Beattie et al. reported inconsistent efficacy (29), and Lai et al. describe improved oxygenation, symptoms, and radiological findings (101). Because these patients typically have more severe pneumonitis and prior immunosuppression, adverse outcomes may reflect indication bias and concomitant infection rather than the drug itself. Principal safety concerns include serious bacterial infection, tuberculosis reactivation, and invasive fungal infection, so active infection should be excluded and latent tuberculosis and hepatitis B screening performed; infliximab should also be avoided in moderate-to-severe immune-related hepatitis or uncontrolled sepsis (11, 29). It is therefore best regarded as a salvage strategy for highly selected patients with a persistent inflammatory phenotype (29, 67, 101).

#### 4.2.3 Tocilizumab

Tocilizumab is a humanized IgG1 monoclonal antibody targeting the interleukin-6 receptor (IL-6R) that blocks IL-6-mediated classical and trans-signaling, potentially attenuating acute inflammation, inflammatory cell recruitment, and Th17-associated responses (102, 103). It has been explored in severe and corticosteroid-refractory CIP and is listed in some guidelines as an escalation option, although evidence derives from case reports, small retrospective studies, and multi-organ irAE cohorts. A single-center retrospective study of 34 patients with corticosteroid-refractory irAEs treated with tocilizumab, of whom 12 had pneumonitis, reported improvement in 27 patients (79.4%) overall but did not report outcomes specific to the pneumonitis subgroup (35). Other data suggest possible improvement in respiratory symptoms and radiological findings, but small samples and absent standardized criteria preclude reliable estimation of response or definition of optimal timing (20, 104). Tocilizumab increases the risk of bacterial, fungal, viral, and mycobacterial infection, and because IL-6R blockade rapidly lowers CRP, a declining CRP cannot exclude infection; no validated IL-6 threshold exists to guide its use in CIP (105, 106).

#### 4.2.4 Intravenous immunoglobulin

IVIG acts through multiple immunomodulatory mechanisms, including competitive binding to Fcγ receptors, modulation of dendritic cell and macrophage function, promotion of regulatory T-cell expansion, inhibition of complement activation, and neutralization of pathogenic autoantibodies. Because it modulates rather than depletes lymphocytes, IVIG may offer potential advantages in patients at high risk of infection or with contraindications to other regimens (107). Evidence for IVIG in corticosteroid-refractory CIP is limited to case reports and small retrospective cohorts. In the retrospective cohort reported by Balaji et al., seven patients received IVIG alone and three received IVIG followed by infliximab; outcomes appeared more favorable among patients receiving IVIG alone, although the small sample size, non-randomized treatment allocation, and differences in disease severity preclude conclusions regarding comparative efficacy (29, 67). Guidelines suggest IVIG may be considered in severe glucocorticoid-unresponsive CIP, commonly at a total dose of 2 g/kg divided over 2–5 days (11).

#### 4.2.5 Cyclophosphamide

Cyclophosphamide is an alkylating cytotoxic immunosuppressant whose active metabolites form DNA cross-links, inhibiting rapidly proliferating T and B lymphocytes (108). Its use in corticosteroid-refractory CIP is largely extrapolated from connective tissue disease-associated interstitial lung disease (109), while CIP-specific evidence is confined to case reports and small studies (29), including combination with tacrolimus in severe CIP after inadequate response to high-dose glucocorticoids, which precludes attribution (110). Given the limited evidence and considerable toxicity, it is generally reserved for critically ill patients in whom other second-line therapies have failed or are unsuitable (9, 10), typically at 1–2 mg/kg/day with individualized dosing (11).

### 4.3 Selected rescue options

#### 4.3.1 Calcineurin inhibition: tacrolimus and cyclosporine

Tacrolimus. Tacrolimus is a calcineurin inhibitor that forms a complex with FK506-binding protein 12, blocking dephosphorylation and nuclear translocation of nuclear factor of activated T cells (NFAT), reducing IL-2 transcription and suppressing T-cell activation (9). Clinical evidence in corticosteroid-refractory CIP derives from case reports and small series: Utsumi et al. reported a patient with recurrent NSCLC and severe CIP that progressed to respiratory failure despite methylprednisolone pulse therapy, in whom tacrolimus and cyclophosphamide added to high-dose glucocorticoids improved symptoms and imaging (110). Because no standardized regimen exists, dosing is extrapolated from transplantation and guided by therapeutic drug monitoring, with attention to renal dysfunction, hypertension, hyperglycemia, neurotoxicity, and CYP3A4-mediated interactions (9).

Cyclosporine. Cyclosporine blocks calcineurin–NFAT signaling and inhibits transcription of IL-2 and other cytokines, and may therefore have value in persistent T cell-mediated inflammation (111, 112). Direct evidence is limited to isolated case reports: in 2024, Deng et al. described a patient with ICI-related pneumonitis who relapsed during tapering and progressed to respiratory failure, in whom adding cyclosporine improved symptoms and imaging and permitted successful tapering (111). Because this single case involved combination therapy, independent efficacy cannot be established and standardized dosing remains undefined.

#### 4.3.2 B-cell-directed therapy: rituximab

Rituximab is a chimeric anti-CD20 monoclonal antibody that induces complement-dependent cytotoxicity, antibody-dependent cell-mediated cytotoxicity, and B-cell apoptosis, resulting in peripheral B-cell depletion. Given the role of B cells in autoantibody production, antigen presentation, and regulation of immune-mediated inflammation, it is widely used in autoimmune diseases (113), with established value in antineutrophil cytoplasmic antibody (ANCA)-associated vasculitis (114). Evidence in corticosteroid-refractory CIP is extremely limited: Moodabagil et al. retrospectively analyzed severe and refractory CIP in which some patients received rituximab, without separately reporting dosing, radiological changes, response, or safety (53). Unlike ANCA-associated vasculitis, CIP has not been shown to feature a B-cell-predominant infiltrate or autoantibody-driven pathogenesis, so rituximab should be considered only on an exploratory basis.

### 4.4 Mechanistically compelling emerging therapies: JAK inhibitors

The JAK–STAT pathway is a key downstream axis through which numerous inflammatory cytokines, including IL-6, IFN-γ, and GM-CSF, regulate immune cell activation, differentiation, and effector function (115, 116). Because these cytokines may contribute to ICI-related inflammation, JAK–STAT targeting has been proposed for refractory irAEs, although evidence that aberrant JAK–STAT activation specifically drives corticosteroid-refractory CIP is insufficient. Tofacitinib, an oral inhibitor principally of JAK1 and JAK3, has been used only experimentally in ICI-related pneumonitis: a 2024 multicenter retrospective study of 53 patients with irAEs included 46 with myocarditis and only two with pneumonitis, without reporting grade, glucocorticoid response, dosing, or individual outcomes (117), and a 2025 prospective cohort suggesting therapeutic potential of JAK inhibitors in refractory irAEs included multiple organ toxicities and agents (75). Guidelines continue to recommend MMF, IVIG, infliximab, tocilizumab, or cyclophosphamide after high-dose glucocorticoid failure, with uniformly low-quality evidence (10). Tofacitinib should therefore be regarded as exploratory, mechanism-directed therapy, used cautiously with infection excluded beforehand and attention to serious infection, thromboembolism, and major adverse cardiovascular events (117, 118), while the theoretical risk of attenuating interferon-dependent antitumor immunity also warrants attention.

### 4.5 Hypothesis-generating therapies

#### 4.5.1 BTK inhibitors: targeting the myeloid–B-cell interface

Bruton tyrosine kinase (BTK) is essential for B-cell receptor signaling and is also expressed in monocytes, macrophages, and neutrophils, participating in Toll-like receptor and Fc receptor signaling and, in some studies, NLRP3 inflammasome activation (119, 120). BTK activation promotes NF-κB-associated inflammatory signaling, regulates IL-1β, IL-6, and TNF-α production, and influences macrophage polarization (119, 121, 122). BTK inhibitors such as ibrutinib, acalabrutinib, and zanubrutinib are widely used in B-cell malignancies and differ in target selectivity (120, 123); *in vitro* and non-CIP models suggest that BTK inhibition may suppress inflammatory cytokine release and attenuate experimental lung injury (121, 122, 124). Because no clinical study has demonstrated efficacy in corticosteroid-refractory CIP, BTK-related signaling is best regarded as a candidate mechanistic research direction.

#### 4.5.2 IL-17 pathway inhibition

The IL-17/Th17 axis promotes neutrophil recruitment, cytokine amplification, and inflammatory lung injury, and contributes to glucocorticoid resistance by enhancing neutrophilic inflammation and interfering with glucocorticoid receptor signaling (9, 57, 125, 126). Plasma and BALF IL-17A levels are significantly elevated in patients with NSCLC who develop CIP and correlate with disease occurrence (57). IL-17A inhibitor such as secukinumab is effective in psoriasis, psoriatic arthritis, and axial spondyloarthritis (127, 128), and case-report and case-series evidence suggests that secukinumab can control ICI-related inflammatory arthritis without evidence of accelerated tumor progression (128). Because no case series, cohort, or prospective study has evaluated IL-17 inhibitors in corticosteroid-refractory CIP, the axis remains a candidate mechanistic target rather than a supported salvage therapy.

#### 4.5.3 Abatacept: restoration of T-cell co-inhibitory signaling

Abatacept is a CTLA-4-Ig fusion protein that binds CD80/CD86 on antigen-presenting cells, blocking CD28-mediated co-stimulation and inhibiting naive T-cell activation. By blocking CD28-mediated co-stimulation, abatacept may have a mechanistic rationale in selected T-cell-mediated irAEs, although its efficacy in CIP and any differential benefit according to ICI class remain unproven. Clinical evidence remains very limited: Salem et al. reported successful use in corticosteroid-refractory immune-related myocarditis, providing indirect support for potential application in severe CIP, but no prospective study has evaluated abatacept in corticosteroid-refractory CIP (129).

### 4.6 Exceptional last-resort therapy: equine anti-thymocyte globulin

Equine anti-thymocyte globulin rapidly depletes T lymphocytes and therefore has mechanistic rationale in critically ill patients with CIP strongly suspected to be T cell-driven that progresses despite glucocorticoids and other immunosuppressants. In 2026, Luong et al. reported the first case of grade 4 CIP treated with equine anti-thymocyte globulin after failure of high-dose intravenous glucocorticoids and MMF, with dosing individualized to peripheral CD2^+^ and CD3^+^ lymphocyte counts; oxygenation and imaging improved, glucocorticoids were tapered, and no relapse occurred over 12 months. Because the evidence is a single case in a patient with melanoma rather than lung cancer, extrapolation to lung cancer-associated CIP remains uncertain (130).

### 4.7 Antifibrotic therapy

Nintedanib inhibits receptor tyrosine kinase signaling through PDGFR, FGFR, and VEGFR, suppressing fibroblast activation and matrix deposition, whereas pirfenidone modulates TGF-β-associated signaling to reduce collagen synthesis (131, 132); both slow lung function decline in idiopathic pulmonary fibrosis, and nintedanib is effective in progressive fibrosing interstitial lung disease (133–135). In CIP, case reports and retrospective studies suggest possible improvement in fibrosis-related radiological changes, but limited samples and non-randomized evidence preclude conclusions regarding efficacy (136, 137). Antifibrotic therapy should not replace immunosuppression for active inflammatory CIP but may be considered an investigational adjunct in selected patients with persistent or progressive fibrotic remodeling.

### 4.8 Gut microbiota-directed intervention

Modulation of the gut microbiota has recently attracted interest as an emerging immunomodulatory strategy in corticosteroid-refractory irAEs, as the microbiota influences immune homeostasis through the gut–lung axis, host immunometabolism, and inflammatory networks (138). Fecal microbiota transplantation (FMT) can remodel microbial composition and modulate the local immune environment: Wang et al. first reported successful FMT in two patients with corticosteroid-refractory ICI-related colitis (139), and Halsey et al. confirmed symptomatic improvement accompanied by microbial remodeling and restored mucosal immune homeostasis (140). Microbiome-directed interventions, particularly adequate dietary fiber intake, may influence ICI efficacy, whereas the benefit of routine probiotic supplementation remains uncertain (138, 141). Direct evidence in corticosteroid-refractory CIP is nevertheless lacking, and because CIP and ICI-related colitis differ substantially in immune composition and injury mechanisms, microbiota-directed strategies require prospective validation.

### 4.9 Combination second-line immunosuppression in corticosteroid-refractory CIP

Despite increasing recognition of corticosteroid-refractory CIP, evidence supporting the combined use of two second-line immunosuppressive agents remains limited and is largely confined to small retrospective cohorts and isolated case reports. No prospective comparative study has established whether dual second-line immunosuppression provides additional benefit over corticosteroids plus a single immunomodulatory agent.

The most direct cohort evidence was reported by Balaji et al., who identified three patients with steroid-refractory CIP treated with IVIG plus infliximab, all of whom died from refractory pneumonitis or infectious complications (67). This should be interpreted cautiously because combination therapy was administered to a particularly critically ill subgroup, in which baseline severity, indication bias, delayed escalation, irreversible lung injury, and concurrent infection may all have contributed. These observations therefore neither prove intrinsic harm nor support routine use, but highlight the difficulty of distinguishing uncontrolled immune-mediated lung injury from infection during profound immunosuppression.

In contrast, several case reports have described favorable outcomes with alternative combination or sequential strategies. Utsumi et al. treated life-threatening pembrolizumab-associated pneumonitis with high-dose corticosteroids plus tacrolimus and cyclophosphamide (110), a regimen carrying substantial risks of opportunistic infection, myelosuppression, and renal toxicity. Ortega Sanchez et al. described immune-related organizing pneumonia that responded to corticosteroids but became resistant to MMF, with improvement after adding infliximab (142), suggesting that sequential targeting of lymphocyte proliferation and TNF-α-mediated inflammation may overcome single-agent resistance in a phenotype that does not generalize to fulminant diffuse alveolar damage. Yu et al. reported recurrent corticosteroid-refractory CIP managed with IVIG and MMF (22), although benefit cannot be attributed to the combination rather than to treatment sequence or natural disease evolution.

Collectively, dual second-line immunosuppression may occasionally serve as an individualized salvage strategy for patients with progressive CIP despite adequate corticosteroid therapy and an initial immunomodulator. The available evidence nevertheless remains anecdotal and highly heterogeneous with respect to radiological phenotype, histopathology, severity, treatment timing, drug sequence, and outcome definitions, and publication bias is likely. No specific dual-agent regimen can therefore be recommended as standard. In particular, phenotypic overlap alone should not be interpreted as an indication for simultaneous multi-agent immunosuppression. Combination immunosuppression should be reserved for carefully selected patients with continued progression despite sequential therapy and should be implemented only through multidisciplinary decision-making, repeated exclusion of infectious mimics, and close surveillance for opportunistic infection.

### 4.10 A proposed phenotype-to-therapy matching framework

The therapeutic evidence reviewed above highlights a central challenge in corticosteroid-refractory CIP: patients who meet a similar clinical definition of glucocorticoid refractoriness may have substantially different underlying biological processes. Current guideline-based management is primarily driven by clinical severity, disease progression, and response to glucocorticoids, whereas the selection of additional immunomodulatory agents remains supported largely by expert consensus, retrospective cohorts, and case reports rather than prospective comparative evidence (7, 10–12, 15, 16, 29, 143). This therapeutic uncertainty is consistent with the mechanistic heterogeneity described in Section 3, where persistent adaptive immune activation, dysregulated cytokine signaling, innate immune activation, and aberrant tissue repair may contribute to corticosteroid refractoriness to different degrees and at different stages of disease. Accordingly, we propose a phenotype-to-therapy matching framework that links putative clinical and biological phenotypes with dominant pathogenic processes and candidate therapeutic directions (**Table 2**). Importantly, this framework is hypothesis-generating rather than prescriptive, because none of the proposed phenotypes or biomarkers has been prospectively validated for selecting a specific second-line therapy.

**Table 2:** Proposed phenotype-to-therapy matching framework for corticosteroid-refractory checkpoint inhibitor pneumonitis.

Putative phenotype	Potential indicators	Dominant biological process	Candidate therapeutic direction	Major limitation
Lymphocyte-dominant inflammatory	BALF lymphocytosis; altered CD4+/CD8+ populations; activated cytotoxic/effector T-cell signatures	Persistent adaptive immune activation	MMF; calcineurin inhibition	No validated biomarker predicting response
Cytokine-dominant hyperinflammatory	Elevated inflammatory mediators, particularly IL-6, in BALF and/or peripheral blood	Persistent cytokine signaling, including IL-6/TNF-alpha-associated pathways	Tocilizumab; infliximab	Cytokine elevation is not an established treatment-predictive marker
Fulminant ALI/DAD-predominant	Rapid hypoxemia; diffuse bilateral GGO; AIP/ARDS-like pattern; ventilatory support	Severe diffuse alveolar injury with overlapping immune pathways	Rapid rescue immunomodulation; IVIG or broader/sequential immunosuppression in selected cases	High mortality; infection and irreversible lung injury confound treatment response
Innate/myeloid-dominant inflammatory	Myeloid remodeling; inflammatory monocyte/macrophage signatures; neutrophilic inflammation	Innate immune activation	Experimental JAK/STAT- or BTK-directed approaches	Predominantly mechanistic/preclinical evidence; no validated treatment-selection criteria
Organizing/fibrotic-remodeling	Persistent reticulation; traction bronchiectasis; architectural distortion or fibrotic progression	Aberrant epithelial repair, macrophage-fibroblast interaction, fibroblast activation and extracellular matrix deposition	Control residual inflammation; consider antifibrotic strategies investigationally	No prospective evidence establishing antifibrotic efficacy or patient selection

This matrix represents a hypothesis-generating conceptual framework rather than a validated clinical algorithm. The proposed phenotypes may overlap and evolve over time, and none of the listed biomarkers has been prospectively validated for selecting a specific second-line therapy. When multiple phenotypes coexist, treatment prioritization should be driven primarily by disease severity and tempo, the likelihood of active infection, the dominant potentially reversible biological process, and agent-specific safety considerations rather than by any single biomarker or radiological pattern.

AIP, acute interstitial pneumonia; ALI, acute lung injury; ARDS, acute respiratory distress syndrome; BALF, bronchoalveolar lavage fluid; BTK, Bruton’s tyrosine kinase; CIP, checkpoint inhibitor pneumonitis; DAD, diffuse alveolar damage; GGO, ground-glass opacity; IVIG, intravenous immunoglobulin; JAK/STAT, Janus kinase/signal transducer and activator of transcription; MMF, mycophenolate mofetil.

One potential phenotype is lymphocyte-dominant inflammatory CIP, characterized by persistent pulmonary lymphocytic inflammation and T-cell activation. BALF studies have demonstrated increased lymphocyte proportions and alterations in CD4^+^ and CD8^+^ T-cell populations in CIP, while single-cell analyses have further revealed activated cytotoxic and effector-memory T-cell states within the pulmonary immune microenvironment (36, 42, 76, 144–146). These findings provide a mechanistic rationale for therapies that suppress lymphocyte proliferation or activation. MMF inhibits lymphocyte proliferation through suppression of *de novo* guanosine nucleotide synthesis, whereas calcineurin inhibitors interfere with T-cell receptor signaling and downstream T-cell activation (99, 112). Nevertheless, neither BALF lymphocytosis nor specific T-cell profiles have been prospectively validated as predictors of response to these agents; therefore, treatment selection based on these features remains investigational.

A second potential phenotype is cytokine-dominant hyperinflammatory CIP, in which sustained inflammatory signaling may contribute to glucocorticoid resistance. Increased concentrations of IL-6 and other inflammatory mediators have been reported in serum or BALF from patients with CIP, supporting the biological plausibility of cytokine-directed therapy (39–41, 57, 144). IL-6 pathway activation provides a rationale for tocilizumab, whereas TNF-α-mediated inflammation provides a theoretical basis for TNF-α blockade with infliximab (35, 65, 66, 101–103, 105). However, neither circulating nor BALF IL-6 or TNF-α concentrations have been validated as treatment-predictive biomarkers in corticosteroid-refractory CIP. Cytokine measurements should therefore currently be regarded as candidate indicators of inflammatory activity or pathway activation rather than established criteria for selecting targeted therapy.

A third clinically important phenotype is fulminant acute lung injury (ALI)/diffuse alveolar damage (DAD)-predominant disease. These patients may present with rapidly progressive hypoxemic respiratory failure, diffuse bilateral ground-glass opacities, an acute interstitial pneumonia/acute respiratory distress syndrome-like radiological pattern, and a need for high-flow oxygen or mechanical ventilation. Histopathological studies have associated ALI/DAD with a more severe clinical course and rapid progression, whereas organizing pneumonia is generally associated with less severe disease (67, 89, 147, 148). Importantly, HRCT findings do not map one-to-one onto histopathology; therefore, DAD cannot be diagnosed solely from an imaging pattern (89, 147). In fulminant disease, several inflammatory pathways may operate simultaneously, providing a rationale for rapid rescue immunomodulation, including IVIG or broader/sequential immunosuppressive approaches in selected patients (29, 67, 107, 110). Nevertheless, treatment response is difficult to interpret because persistent deterioration may reflect uncontrolled inflammation, irreversible alveolar injury, superimposed infection, or a combination of these processes.

A fourth potential phenotype is innate immune- or myeloid-dominant inflammation. BALF and single-cell studies have demonstrated remodeling of pulmonary myeloid populations in CIP, including inflammatory monocyte/macrophage populations, while neutrophilic inflammation may be prominent in selected patients (42, 76, 84). These findings are consistent with the potential involvement of macrophage activation, neutrophil-associated pathways, and innate immune signaling in persistent pulmonary injury. Mechanistically, JAK/STAT signaling represents a potential therapeutic target for modulating inflammatory signaling and myeloid-cell activation (72, 73). However, evidence supporting these approaches specifically in corticosteroid-refractory CIP remains extremely limited, and they should currently be regarded as investigational rather than established rescue strategies.

A fifth phenotype may emerge when the dominant pathological process shifts from active inflammation toward organizing or fibrotic remodeling. Persistent epithelial injury, dysfunctional alveolar regeneration, macrophage–fibroblast interactions, fibroblast activation, and extracellular matrix deposition may contribute to incomplete resolution and progressive structural lung damage (51, 52, 78, 91–97). Radiological evidence of persistent reticulation, traction bronchiectasis, architectural distortion, or progressive fibrotic change may raise concern for this transition, although imaging alone cannot reliably determine whether active immune inflammation remains present. This distinction is clinically important because repeated escalation of immunosuppression may provide diminishing benefit once structural remodeling becomes dominant. In selected patients with progressive fibrotic change, antifibrotic agents such as nintedanib or pirfenidone have been explored; however, evidence in corticosteroid-refractory CIP remains limited to case reports, case series, and retrospective observations and does not support routine use (131–137).

The proposed phenotypes are neither mutually exclusive nor temporally fixed. A patient may simultaneously exhibit lymphocyte activation, cytokine dysregulation, innate immune inflammation, and acute alveolar injury, and the dominant pathological process may change during the disease course. Moreover, BALF cellular profiles and inflammatory biomarkers may be influenced by concurrent infection, underlying lung disease, sampling timing, and prior antimicrobial or glucocorticoid exposure (36, 40, 50, 76, 145). Consequently, a single radiological pattern, BALF parameter, or circulating biomarker is unlikely to provide sufficient biological resolution for treatment selection. The proposed phenotype-to-therapy framework and the principal limitations of each therapeutic direction are summarized in **Table 2**.

When phenotypic features overlap, therapeutic prioritization should not depend on assigning the patient to a single category. Instead, we suggest a pragmatic hierarchy based on four considerations: disease severity and tempo, the probability of active infection, the dominant reversible biological process, and the toxicity profile of the candidate rescue agent. In rapidly progressive hypoxemic disease or an ALI/DAD-like presentation, immediate stabilization and timely rescue immunomodulation should take precedence over precise phenotypic classification. If infection cannot be confidently excluded, escalation to strongly immunosuppressive cytokine-directed or cytotoxic therapies should be approached cautiously, while microbiological evaluation and empiric antimicrobial therapy are pursued as clinically appropriate. In patients with mixed lymphocytic and cytokine-dominant inflammation, the choice between MMF, calcineurin inhibition, or cytokine-directed therapy should be guided by the most compelling clinical and biological evidence rather than by any single biomarker, because none has been validated as treatment predictive. Conversely, when serial HRCT and clinical evolution suggest increasing organization or fibrotic remodeling with diminishing evidence of active inflammation, repeated escalation of immunosuppression may offer decreasing benefit and increasing harm. Thus, the proposed framework should be applied dynamically, with the dominant and most immediately reversible process reassessed after each therapeutic step.

Taken together, this framework reframes the therapeutic question from “which second-line agent is generally preferred?” to “which biological process is dominant in a given patient at a given stage of disease, and which intervention is most likely to modify that process?” Translating this conceptual framework into clinical practice will require dynamic, multimodal phenotyping and prospective validation of treatment-predictive biomarkers. These requirements form the basis of the emerging precision-management strategies discussed below.

### 4.11 Comparative safety and infection-related treatment selection

The safety profiles of second-line therapies are particularly important in corticosteroid-refractory CIP because clinical deterioration may reflect persistent immune-mediated pneumonitis, superimposed infection, or both, and prolonged corticosteroid exposure itself increases susceptibility to opportunistic infection. Available studies do not permit a formal ranking of second-line agents by safety or therapeutic index, because treatment selection is strongly confounded by baseline severity, indication bias, prior immunosuppression, and concomitant infection. Nevertheless, several clinically relevant distinctions can be made.

IVIG is immunomodulatory rather than broadly lymphocyte-depleting and may therefore be considered when additional profound immunosuppression is undesirable or when infection risk is particularly high. However, it should not be regarded as uniformly “safer,” because infusion reactions, thrombosis, hemolysis, renal dysfunction, and volume overload remain important concerns. MMF provides broader suppression of lymphocyte proliferation and has a slower onset of action; infection, cytopenias, gastrointestinal toxicity, and hepatotoxicity require monitoring, making it less attractive as sole rescue therapy in rapidly deteriorating patients. Infliximab carries clinically important risks of serious bacterial, fungal, and mycobacterial infection and should generally be avoided in uncontrolled infection or sepsis; tuberculosis and hepatitis B screening are also required when feasible. Tocilizumab similarly increases susceptibility to infection and may complicate infection surveillance because IL-6 receptor blockade rapidly suppresses CRP, such that a falling CRP level cannot reliably exclude ongoing infection. Cyclophosphamide and calcineurin inhibitors provide broader immunosuppression and carry substantial toxicity, including myelosuppression, infection, renal toxicity, and other organ-specific adverse effects, and are therefore best reserved for highly selected rescue situations.

Accordingly, when occult infection cannot be confidently excluded, no second-line agent can be considered completely safe. A reasonable risk-minimizing approach is to obtain lower respiratory tract microbiological sampling whenever feasible, initiate or continue appropriate antimicrobial coverage when clinically indicated, and avoid premature escalation to agents with profound or prolonged immunosuppressive effects. In this setting, IVIG may have a relative practical advantage because it does not produce the same degree of targeted cytokine blockade or lymphocyte suppression, but the evidence is insufficient to establish superior safety or efficacy. Once infection has been reasonably excluded and a persistent inflammatory phenotype remains likely, the choice of MMF, infliximab, tocilizumab, or another agent should be individualized according to disease tempo, suspected dominant pathway, comorbidities, previous immunosuppression, and agent-specific contraindications.

## 5 Emerging precision-management strategies

The phenotype-to-therapy framework proposed above provides a conceptual model for interpreting biological heterogeneity in corticosteroid-refractory CIP, but it cannot yet be used as a clinical treatment algorithm. The key translational challenge is therefore no longer simply to identify additional immunosuppressive agents, but to develop tools capable of determining the dominant disease process, tracking its evolution over time, and predicting response to specific therapeutic interventions. Three complementary directions are particularly relevant: development of treatment-predictive biomarkers, longitudinal molecular characterization of disease states, and integration of multidimensional data through computational approaches.

### 5.1 From candidate biomarkers to treatment-predictive biomarkers

The most immediate requirement for precision management is to distinguish biomarkers of disease presence or severity from biomarkers that actually predict response to a specific therapy. Serum and BALF studies have identified alterations in inflammatory cytokines, chemokines, lymphocyte subsets, and other immune-cell populations in CIP (36, 39–42, 144–146). Similarly, serum markers such as KL-6 and radiological features may contribute to disease characterization or prognostic assessment (33, 34, 148). However, association with severe disease does not establish therapeutic predictiveness.

For example, increased IL-6 concentrations provide biological support for IL-6 pathway involvement, but there is currently no prospective evidence that patients with higher IL-6 levels derive greater benefit from tocilizumab (35, 39, 41, 105, 149). The same limitation applies to BALF lymphocytosis, altered CD4^+^/CD8^+^ T-cell proportions, neutrophilic inflammation, and other pulmonary immune profiles. These features may reflect local immune activity but are not specific to corticosteroid-refractory CIP and may be affected by infection, underlying lung disease, treatment exposure, and sampling time (36, 40, 50, 76, 145, 146). At present, therefore, no circulating, BALF, imaging, or molecular marker has been prospectively validated for selecting MMF, infliximab, tocilizumab, IVIG, or another second-line agent.

Future biomarker studies should consequently be embedded within prospective therapeutic cohorts rather than conducted solely as cross-sectional comparisons between CIP and control populations. Biomarkers should be measured before treatment escalation, sampled longitudinally where feasible, and analyzed against drug-specific response endpoints. Demonstrating that a marker is associated with outcome is insufficient; clinically useful predictive biomarkers should identify differential probability of response to a particular intervention. This shift from prognostic association to treatment–biomarker interaction is essential if the phenotype-to-therapy framework proposed in Section 4.10 is to become clinically actionable.

### 5.2 Longitudinal single-cell, spatial, and multi-omics profiling

Single-cell RNA sequencing (scRNA-seq) and related technologies offer an opportunity to define the cellular states underlying conventional clinical phenotypes. BALF single-cell studies have demonstrated substantial immune remodeling in CIP, including activation of T-cell populations, enrichment of cytotoxic lymphocytes, and alterations in inflammatory monocyte/macrophage compartments (25, 42, 84). Conventional bulk measurements may obscure such cellular heterogeneity, whereas single-cell approaches can identify distinct immune populations and signaling programs potentially associated with persistent pulmonary inflammation.

For corticosteroid-refractory disease, however, longitudinal profiling may be more informative than increasingly detailed cross-sectional characterization. A single sample obtained during established pneumonitis cannot determine whether an observed immune state is transient, steroid-sensitive, persistently activated despite glucocorticoid exposure, or part of a transition toward tissue remodeling. Serial sampling before and after glucocorticoid and second-line therapy could help identify cellular programs associated with treatment response, persistence, or progression.

Spatial transcriptomic approaches may further define how activated lymphocytes and myeloid populations interact with alveolar epithelial cells, endothelial cells, and fibroblasts within injured lung tissue. This may be particularly relevant to the proposed transition from inflammatory injury toward maladaptive repair and fibrosis, for which experimental and pulmonary fibrosis studies implicate persistent transitional epithelial states, monocyte-derived macrophages, and profibrotic epithelial–mesenchymal interactions (51, 52, 78, 91–97). Integration of transcriptomics with proteomics, metabolomics, T-cell receptor clonality, BALF immunophenotyping, and longitudinal clinical outcomes may ultimately permit identification of reproducible molecular endotypes associated with glucocorticoid refractoriness and differential treatment response (25, 42, 76, 150).

These technologies should nevertheless remain framed as mechanistic discovery and biomarker-development tools, rather than current clinical decision instruments. Their translational value will depend on whether molecular signatures identified in discovery cohorts can be reproduced across centers and, more importantly, whether they predict clinically meaningful responses to specific therapeutic interventions.

### 5.3 AI-assisted multimodal decision support

Because corticosteroid-refractory CIP is biologically heterogeneous and dynamically evolving, future decision support may benefit from integrating serial clinical, imaging, biomarker, and treatment-response data rather than relying on any single marker. Radiomics and machine-learning approaches have already been explored for CIP detection, differential diagnosis, and risk stratification, including discrimination of ICI-related pneumonitis from radiation pneumonitis (151, 152). However, their application to corticosteroid-refractory CIP remains highly preliminary, and no AI-based model has been validated to guide rescue-therapy selection or predict treatment response. Any future AI-assisted system should therefore be viewed as a research tool that complements, rather than replaces, multidisciplinary clinical judgment. Progress will depend on standardized case definitions, longitudinal multimodal datasets, treatment-specific outcomes, external validation, and prospective clinical evaluation before such models can be considered for therapeutic decision support (153).

## 6 Discussion and future perspectives

Despite increasing recognition of corticosteroid-refractory CIP, major uncertainties remain in its definition, biological classification, treatment sequencing, and long-term management. The central challenge is to move from empiric escalation of immunosuppression toward prospective, mechanism-informed care while minimizing infection, treatment toxicity, and unnecessary impairment of antitumor immunity. Progress will require harmonized definitions, longitudinal phenotyping, comparative therapeutic evidence, and clinically relevant outcome measures.

### 6.1 Standardization and comparative clinical evidence

A universally accepted operational definition of corticosteroid-refractory CIP remains lacking. Future studies should standardize the glucocorticoid regimen, timing of response assessment, objective criteria for treatment failure, and distinction among primary refractoriness, delayed response, steroid dependence, and recurrence. Core outcomes should include oxygen and ventilatory requirements, radiological response, glucocorticoid exposure, recurrence, infectious complications, and mortality. Comparative studies should evaluate not only which second-line agent is used, but also treatment timing and sequence. Because randomized trials may be difficult in this rare and severe condition, multicenter prospective registries with harmonized clinical variables, serial biosampling, and predefined outcomes should be prioritized. These platforms could support pragmatic or adaptive studies while reducing confounding from baseline severity, infection, prior immunosuppression, and treatment delay.

### 6.2 Dynamic phenotyping and treatment stratification

A central unresolved issue is whether persistent abnormalities after glucocorticoid therapy reflect ongoing immune inflammation, evolving structural lung injury, or both. Because these processes may coexist and change over time, future classification should be dynamic rather than based on a single baseline phenotype. Serial HRCT, oxygen requirement, pulmonary function, BALF cellular profiles, circulating inflammatory markers, and molecular data may help define changes in the dominant disease process. Biomarker research should therefore shift from diagnostic association toward treatment stratification. Candidate biomarkers and molecular endotypes should be assessed prospectively before and after treatment escalation and tested for their ability to predict differential therapeutic response. The phenotype-to-therapy framework proposed in this review should be regarded as a testable model that requires external validation rather than as a validated clinical algorithm.

### 6.3 Safety, rechallenge, and long-term outcomes

Treatment escalation must balance control of pulmonary inflammation against opportunistic infection, treatment-related toxicity, and potential effects on antitumor immunity. Future studies should therefore report infectious complications and oncological outcomes alongside pulmonary endpoints and evaluate the overall therapeutic index of rescue strategies rather than short-term pneumonitis response alone. Long-term management also requires better evidence for ICI rechallenge and pulmonary recovery. Prospective cohorts should evaluate recurrent CIP, chronic radiological abnormalities, pulmonary function, glucocorticoid dependence, quality of life, cancer control, and survival. Whether the initial biological phenotype or degree of structural lung injury can inform rechallenge risk remains an important but currently unvalidated question.

### 6.4 Research roadmap and conclusion

A practical research roadmap should proceed in three stages. First, the field should establish harmonized definitions, outcome measures, and multicenter prospective registries with standardized longitudinal sampling. Second, these datasets should be used to identify and externally validate clinically measurable disease states and treatment-relevant endotypes. Third, prospective studies should test whether mechanism-informed treatment selection improves clinical outcomes compared with empiric escalation. Emerging technologies such as multi-omics and AI should be evaluated only insofar as they improve disease-state classification, prediction of therapeutic response, or patient outcomes. Ultimately, the goal is not to create increasingly complex taxonomies, but to determine whether the biological heterogeneity of corticosteroid-refractory CIP can be translated into safer, more effective, and clinically actionable treatment strategies.

## 7 Conclusion

Corticosteroid-refractory checkpoint inhibitor pneumonitis represents one of the most severe and therapeutically challenging immune-related adverse events associated with ICI therapy. Current evidence suggests that it should not be regarded simply as a more severe form of conventional CIP or as a uniform failure of glucocorticoid treatment, but rather as a biologically heterogeneous and dynamically evolving syndrome in which persistent adaptive immune activation, dysregulated cytokine signaling, innate immune abnormalities, and maladaptive tissue repair contribute to different degrees at different stages.

This heterogeneity provides a plausible explanation for the inconsistent outcomes observed with empirical second-line immunosuppression. Although MMF, infliximab, tocilizumab, IVIG, cyclophosphamide, calcineurin inhibitors, and other rescue strategies have been used in practice, current evidence remains insufficient to establish a universally preferred agent or treatment sequence. Careful exclusion of infection, assessment of clinical and radiological trajectory, and multidisciplinary individualized decision-making therefore remain fundamental, and persistent disease after glucocorticoid therapy may not always reflect ongoing immune inflammation, since progressive structural lung injury may increasingly determine outcome.

The next phase of research should move beyond empirical escalation toward prospectively validated, mechanism-informed management. Harmonized definitions, standardized response criteria, international prospective cohorts, longitudinal biological sampling, and comparative therapeutic studies will be essential to determine whether reproducible clinical and molecular endotypes can predict treatment response. In particular, distinguishing persistent immune-mediated inflammation from disease that has transitioned toward structurally entrenched lung injury may become a critical basis for selecting therapeutic intensity and avoiding ineffective immunosuppression. The central goal is ultimately not to identify a more potent rescue agent, but to determine which biological process is driving refractoriness in a given patient at a given stage of disease and to match treatment to that process.
